# The Parkinsonian Subthalamic Network: Measures of Power, Linear, and Non-linear Synchronization and their Relationship to L-DOPA Treatment and OFF State Motor Severity

**DOI:** 10.3389/fnhum.2016.00517

**Published:** 2016-10-25

**Authors:** Timothy West, Simon Farmer, Luc Berthouze, Ashwani Jha, Martijn Beudel, Thomas Foltynie, Patricia Limousin, Ludvic Zrinzo, Peter Brown, Vladimir Litvak

**Affiliations:** ^1^Centre for Mathematics and Physics in the Life Sciences and Experimental Biology, UCLLondon, UK; ^2^Wellcome Trust Centre for Neuroimaging, UCL Institute of NeurologyLondon, UK; ^3^Department of Neurology, National Hospital for Neurology and NeurosurgeryLondon, UK; ^4^Sobell Department of Motor Neuroscience and Movement Disorders, Institute of Neurology, UCLLondon, UK; ^5^Centre for Computational Neuroscience and Robotics, University of SussexFalmer, UK; ^6^UCL Great Ormond Street Institute of Child Health, UCLLondon, UK; ^7^Department of Neurology, University Medical Center Groningen, University of GroningenGroningen, Netherlands; ^8^Nuffield Department of Clinical Neurosciences, John Radcliffe HospitalOxford, UK; ^9^Medical Research Council Brain Network Dynamics Unit, University of OxfordOxford, UK

**Keywords:** Parkinson's disease, deep brain stimulation (DBS), connectivity, coherence, detrended fluctuation analysis, synchronization, criticality

## Abstract

In this paper we investigated the dopaminergic modulation of neuronal interactions occurring in the subthalamic nucleus (STN) during Parkinson's disease (PD). We utilized linear measures of local and long range synchrony such as power and coherence, as well as Detrended Fluctuation Analysis for Phase Synchrony (DFA-PS)- a recently developed non-linear method that computes the extent of long tailed autocorrelations present in the phase interactions between two coupled signals. Through analysis of local field potentials (LFPs) taken from the STN we seek to determine changes in the neurodynamics that may underpin the pathophysiology of PD in a group of 12 patients who had undergone surgery for deep brain stimulation. We demonstrate up modulation of alpha-theta (5–12 Hz) band power in response to L-DOPA treatment, whilst low beta band power (15–20 Hz) band-power is suppressed. We also find evidence for significant local connectivity within the region surrounding STN although there was evidence for its modulation via administration of L-DOPA. Further to this we present evidence for a positive correlation between the phase ordering of bilateral STN interactions and the severity of bradykinetic and rigidity symptoms in PD. Although, the ability of non-linear measures to predict clinical state did not exceed standard measures such as beta power, these measures may help identify the connections which play a role in pathological dynamics.

## Introduction

Synchronized activity in the brain facilitates long distance communication and sensory integration (Salinas and Sejnowski, 2001; Buzsáki and Draguhn, 2004; Fries, 2015). It has been proposed that synchronization in the brain is delicately poised at a transition between completely ordered and disordered interactions (Kitzbichler et al., 2009; Botcharova et al., 2014), a feature that may arise more generally from brain dynamics that are self-organized at the edge of stability (Beggs, 2008; Shew et al., 2009; Chialvo, 2010). The so called *metastable* dynamics that arise at this transition, are hypothesized to facilitate optimal information transfer (Barnett et al., 2013), maximize dynamic range and adaptability (Kinouchi and Copelli, 2006; Shew et al., 2009) as well as increase the network's capacity for information storage (Shew et al., 2011). In the case of pathological states such as epilepsy or tremor, strong neural synchrony that is resistant to external perturbation impairs network function (McAuley, 2000; Schnitzler and Gross, 2005; Uhlhaas and Singer, 2006; Hammond et al., 2007; Hirschmann et al., 2013). Persistent oscillations are indicative of limit cycle behaviour- the inability to break from this regime may impair the brain's ability to desynchronize, a feature proposed to be important to information coding (Benda et al., 2006; Hanslmayr et al., 2012) and motor performance (Kühn et al., 2004).

In the motor network of healthy human subjects there are weak beta band oscillations that favor maintenance of steady muscle contraction. These are readily suppressed during movement and also during movement observation (Farmer et al., 1993; Conway et al., 1995; Baker et al., 1999). Increased beta band (15–35 Hz) oscillations in the local field potential (LFP) recorded from the basal ganglia during functional neurosurgery (deep brain stimulation–DBS) have been reliably observed to be a hallmark of dopaminergic depletion and akinesia in Parkinson's Disease (Brown et al., 2001; Kühn et al., 2006; Weinberger et al., 2006; Hammond et al., 2007).

The finding of excessive beta oscillations in PD may imply a shift from metastability to stability that occurs in the onset of neurological disease (Dotov, 2014). It has been suggested that these dynamics produce a reduction in the information encoding space available to the subcortical motor network that result in the akinetic symptoms of PD (Hanslmayr et al., 2012; Brittain and Brown, 2014). The mechanisms that generate beta oscillations in subcortical networks are unknown although several models have been suggested that involve either increased cortical drive to a subcortical resonator or a change in cortical feedback (Pavlides et al., 2015). More complete descriptions of network dynamics will help to discriminate between these potential models.

In this paper, we characterize neural dynamics of local field potentials recorded from different electrode contacts within bilateral subthalamic nuclei (STN) in Parkinson's patients undergoing DBS surgery. As the recording electrode contacts are not entirely enclosed within STN we describe our results in terms of signals originating from the region surrounding the STN which we abbreviate STNr. The recordings were done in two medication states: ON and OFF Levodopa (L-DOPA). We analyze local STNr power estimates along with measures of functional connectivity within and between the two STNrs.

For excessive oscillations to affect information processing in a way that influences the capacity for normal movement then they should have detectable correlates in measures of network synchrony. We choose to quantify this synchrony through statistical measures of dependencies between signals which we hereon term functional connectivity. We use a linear measure of the correlation between neurophysiological time series- spectral coherence, as well as weighted phase lag index (WPLI) (Vinck et al., 2011) which we compare with coherence as it has been demonstrated to be robust to zero-lag volume conduction effects—a potential confound when recordings are made in close spatial proximity. We hypothesize that connectivity within and between the STNr(s) is significantly altered in PD and that we can infer changes in the strength of this functional connectivity through measures of statistical dependency between signals.

In addition to the above approach, we utilize a recently developed measure that characterizes the temporal ordering of phase dynamics between weakly coupled signals using Detrended Fluctuation analysis for Phase Synchrony (DFA-PS, (Botcharova et al., 2014)). Autocorrelation length of fluctuations has been proposed as a possible statistical tool to approximate a system's proximity to a super-critical Hopf bifurcation (Aburn et al., 2012) and is known to be a signature of critical transitions in general (Scheffer et al., 2009). By using DFA-PS it is possible to detect changes in the scaling statistics of phase interactions. Previous modeling work has suggested that scaling of phase synchrony interactions may be a proxy measurement of a system's propensity to synchrony (Kitzbichler et al., 2009; Botcharova et al., 2014). As excess synchrony in the STN is well known to be a hallmark of dopaminergic depletion, we hypothesize that autocorrelation in the phase dynamics, estimated from the slope of the DFA plot will be greater in untreated PD. This may indicate that system may lay closer to a critical transition beyond which there is greater neural synchrony.

We further assess the changes in the physiological measures for basal ganglia dynamics that occur with administration of L-DOPA in the PD resting state. We study correlations between changes in the physiological parameters and the severity of motor impairment with respect to measures of Parkinsonian bradykinesia and rigidity. In particular, we are interested in whether the application of non-linear measures of synchronization (DFA-PS) provides additional information that allows better characterization of patients' clinical state over that of more conventionally used signal processing methods such as power and coherence.

## Materials and methods

### Experimental setup

#### Patient details and surgical implantation of DBS electrodes

The majority of the data were taken from a study involving a 17 patient cohort who had all undergone surgery for chronic implantation of DBS electrodes in the STN (see Litvak et al., 2011, 2012). The patients have undergone simultaneous magnetoencephalography (MEG) and intracranial recordings, but here we focus on intracranial data only. The data included in the present analysis are from 12 patients in whom bilateral STN-LFP recordings were done in both ON or OFF dopaminergic medication state. This cohort comprises 9 patients from the original study plus another 3 from another study not previously reported. The selected cohort's clinical details are summarized in Table 1. The study was approved by the joint ethics committee of the National Hospital for Neurology and Neurosurgery and the UCL Institute of Neurology, and the patients gave written informed consent before the study onset.

**Table 1:** **Details of DBS patient cohort**.

**Patient**	**Age (years)**	**Sex**	**Disease duration (years)**	**Predominant symptoms**	**Motor UPDRS (ON/OFF)**	**Pre-operative medication**
1	58	M	13	Gait freezing, dyskinesias, pain	25/43	Co-careldopa 1000 mg
						Co-careldopa modified release 125 mg
						Amantadine 400 mg
						Co-beneldopa 125 mg
						Entacapone 600 mg
						Rasagiline 1 mg
2	57	M	17	Gait impariment, pain, dyskinesias	14/54	Co-careldopa 1125 mg
						Co-careldopa modified release 250 mg
						Co-beneldopa 200 mg
						Entacapone 1600 mg
						Selegiline 10 mg
						Amantadine 200 mg
3	60	M	15	Dyskinesias, gait freezing, bradykinesia, tremor	10/56	Co-careldopa 1125 mg
						Co-beneldopa 250 mg
						Ropinirole 18 mg
						Selegiline 10 mg
						Amantadine 200 mg
4	48	M	11	Gait freezing, tremor	16/72	Rasagiline 1 mg
						Co-careldopa 1250 mg
						Entacapone 500 mg
5	52	M	12	Dystonia, motor fluctuations, tremor	10/35	Rotigotine 4 mg
						Stalevo 950 mg
						Rasagiline 1 mg
6	58	F	10	Dystonia, dyskinesias, tremor	16/55	Pramipexole 3 mg
						Stalevo 400 mg
						Rasagiline 2 mg
						Co-beneleldopa 62.5 mg as required
7	55	M	15	Tremor, freezing	5/19	Co-beneldopa 1000 mg
						Ropinirole16 mg
						Selegiline10 mg
						Amantadine100 mg
8	54	M	8	Gait impariment, dyskinesias, tremor	18/51	Cabergoline 4 mg
						Entacapone 800 mg
						Co-careldopa 1200 mg
						Amantadine 300 mg
9	58	F	14	Gait freezing, pain, dyskinesias, tremor	16/55	Pramipexole 4 mg
						Stalevo 250 mg
10	53	M	17	Motor fluctuations, dyskinesias, freezing	9/35	Sinemet 750 mg
						Pramipexole 0.54 mg
11	60	F	20	Tremor, dyskinesias, gait impairment	4/35	Sinemet CR 200 mg
						Sinemet 563 mg
						Stalevo 300 mg
						Rasagiline 1 mg
12	53	M	12	Tremor, freezing, motor fluctuations, gait impairments	19/53	Stalevo 750 mg
						Amantidine 100 mg
						Pramipexole 2.64 mg

All patients have PD and have been treated with deep brain stimulation (DBS) targeted to the STN. The patients' age, gender, duration of PD since diagnosis, predominant clinical features, unified Parkinson's disease rating scale (UPDRS, 2008 part III- Motor Impairment) ON and OFF medication are shown. The score is out of a maximum of 104 with higher scores indicative of increased severity.

All patients were diagnosed with PD according the Queen Square Brain Bank criteria (Gibb and Lees, 1988). The selection criteria, operative procedure and clinical outcomes of DBS therapy have been previously reported (Foltynie et al., 2011). The degree of clinical impairment was assessed prior to surgery (<5 months preoperatively) using part III (motor impairment) of the Unified Parkinson's Disease Rating Scale (UPDRS) following overnight withdrawal of all dopaminergic medication (OFF) and following administration of their pre-operative dose of levodopa (ON). This gave a wide range of UPDRS scores by which we could correlate signal features with the degree of patients' motor impairment.

All patients had a pre-operative stereotactic MRI using T2-weighted with fast acquisition images. The subthalamic target was visualized on MRI and directly targeted using planning software (FrameLink4TM, Version 2003, Medtronic, Minneapolis, MN). All patients were implanted bilaterally with a quadripolar DBS electrode (Model 3389 DBS, lead, Medtronic). This surgical procedure has been described previously (Zrinzo et al., 2009; Foltynie et al., 2011). Postoperative stereotactic MR were imported into the planning software allowing 3-dimensional reconstruction of the images along the electrode trajectory (Framelink, Medtronic; Holl et al., 2010). The coordinates of each contact were transposed onto the pre-operative stereotactic MRI (Hariz et al., 2003).

For each contact it was determined whether it was (i) inside, at the border (ii) or outside (iii) of the STN. The relation to the STN was assessed on both axial and coronal MRI sections. In case the contact was inside the STN, a two was assigned to the contact, in case the contact was at the border of the STN, a one was assigned to the contact and if the contact was outside the STN, a zero was assigned to the contact. Given the axial and coronal sections this resulted in a score between zero and four per contact. In case the total score was zero or one the contact was considered to be outside the STN, in case the total score was two the contact was considered to be at the border of the STN and in case the total score was three or four the contact was considered to be inside the STN. After implantation, electrodes were connected to an accessory kit, typically with both connectors being tunneled to the left temporo-parietal area and externalized through the frontal region. No microelectrode recordings were made.

The patients were studied in the interval between DBS electrode implantation and subsequent connection to a subcutaneous stimulator between 2 and 7 days postoperatively. This allowed for STN-LFP to be recorded and our data was collected during this period.

##### Recording setup

Local field potentials and electromyographic (EMG) signals were simultaneously collected using an EEG device integrated in the MEG scanner (CTF/VSM MedTech, Coquitlam, BC, Canada). Bipolar EMG recordings were made from the muscle belly of the first dorsal interosseous of the hand referenced to the tendon. Signals were hardware high-pass filtered (>1 Hz) in order to avoid saturation of the amplifier resulting from large DC offsets. In addition head location data were continuously collected using the Head Position Indicator (HPI) coils placed on the subject's nasion and the two pre-auricular points. The data were sampled at 2400 Hz and stored to disk.

**Figure 1:**
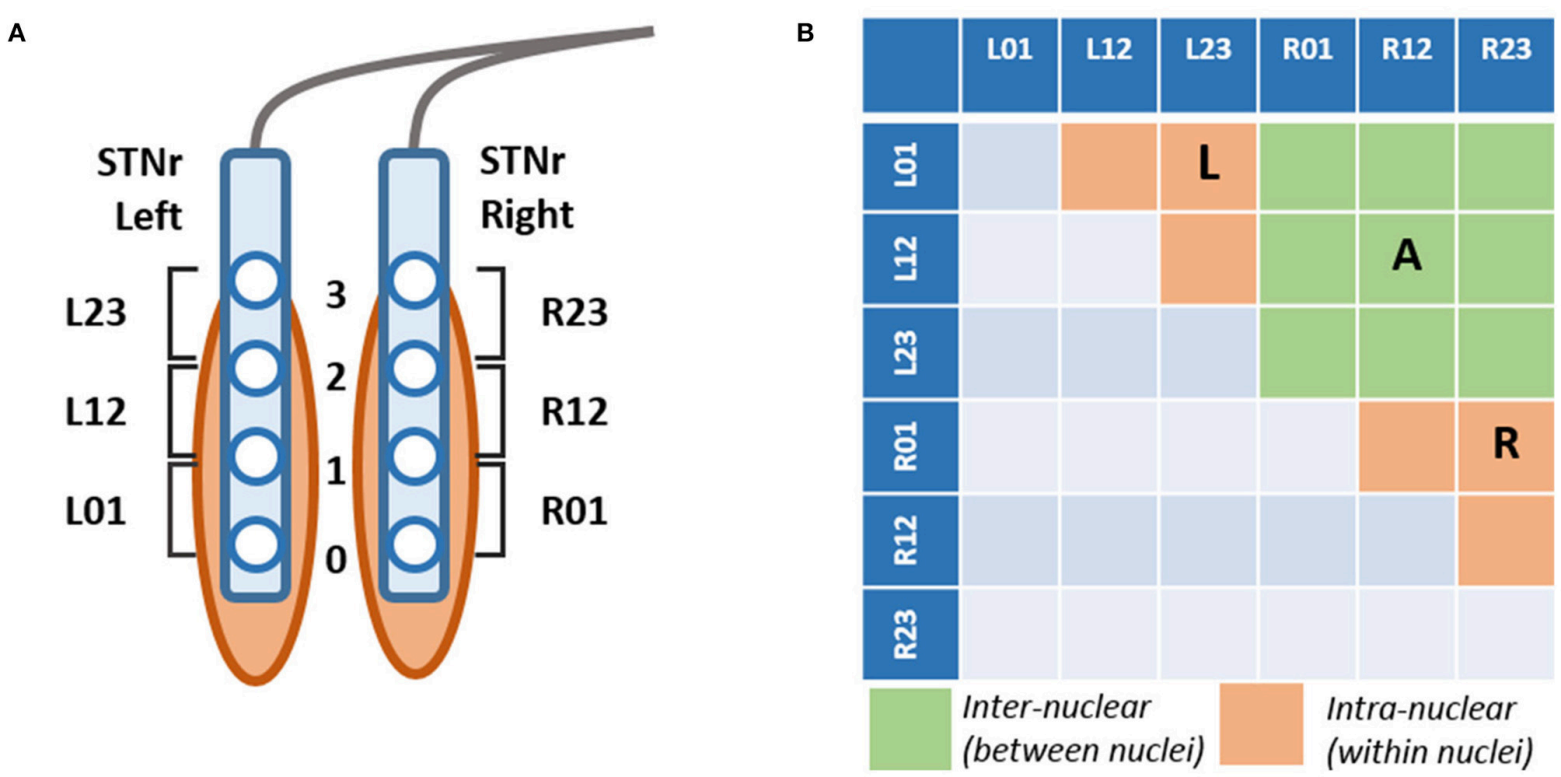
**(A)** Schematic of DBS electrode contact placement respective the STN and bipolar derivation of channels. Each electrode comprises 4 contacts montaged to 3 bipolar channels Left/Right 01,12,23 STNr. Contact 0 is targeted to be 2 mm caudal of the center of the STN. **(B)** Derivation of channel pairs subjected to neurophysiological connectivity analyses. Channel pairings were divided into those within the hemisphere (intra-nuclear, in orange) or between left and right nuclei (inter-nuclear, in green). The intra-nuclear pairs were separated into two separate groups corresponding to those originating from left (L) and right (R) nuclei. Not all contacts are contained within the STN and thus signals are referred to as originating from within the local region of the nuclei which we abbreviate STNr.

The STN electrodes have four contacts at 0,1,2,3 where the zero contact is targeted 2 mm below the center of the STN. LFP recordings were made with reference to a cephalic reference electrode and later converted offline to a bipolar montage giving rise to 3 channels consisting each of a pair of adjacent contacts (see Figure 1).

#### Experimental paradigm

Two recordings were obtained for each patient: one recording was performed after overnight withdrawal (>10 h) from dopaminergic medication, termed the OFF state; the other recording was obtained in the ON state in which the patient had taken their usual dose of medication (>200 mg of levodopa). The ON and OFF recordings were done on different days and the order was counterbalanced across patients.

Data presented here were taken from an experiment in which recordings were made from patients sitting comfortably upright in a state of wakeful rest with their hands on the chair armrests. Subjects were visually cued using MATLAB (The MathWorks, Inc., Natick, Massachusetts) and the Cogent (http://www.vislab.ucl.ac.uk/cogent.php) toolbox and instructed to remain motionless with their eyes open and focused on a fixation cross. The recordings were made for approximately 3 min in the presence of a neurologist who monitored task performance and patient's wellbeing. None of the patients developed tremor during the recordings; their dominant symptoms were bradykinesia and rigidity. EMG was inspected for each patient in the rest period to identify any large/regular movements occurring during the recording. None were found and so a motor rest state was confirmed.

### Signal analysis

#### Analysis software

SPM12 (release v6685, http://www.fil.ion.ucl.ac.uk/spm/) was used for data conversion, referencing and Parametric Empirical Bayes analyses. Fieldtrip (contained within SPM release, http://www.ru.nl/neuroimaging/fieldtrip/) was used for preprocessing, spectral and connectivity analyses. Regression analyses and mixed modeling were computed using IBM's SPSS software (SPSS Statistics V 22.0, IBM Corp., Armonk, NY). All other analyses, statistics and plotting were computed using proprietary MATLAB scripts.

#### Pre-processing of LFP recordings

Prior to analysis, we inspected the raw LFP recordings in order to identify irregularities and artifacts in the recordings. One patient was removed from the study due to data loss in the left STN channels resulting from amplifier saturation preventing further analysis. All other recordings were deemed valid for further pre-processing and analysis giving a total number of 12 patients included in the rest of the analyses.

Application of DFA requires continuous data and for these analyses we opted for a conservative scheme of pre-processing where we aimed (if possible) to rectify artifacts rather than remove them entirely.

Large amplitude jumps in the recordings that spanned multiple channels of a trial were obvious from visual inspection. These jumps originate from transient discharges in the amplifier that could not be entirely resolved at the hardware level. In order to correct for this artifact we applied a threshold detection method such that when the amplitude exceeded 3 standard deviations the corresponding region of recording was removed. Resulting gaps in the time series were infrequent and transient (5–15 occurrences per recording, 1–20 samples in the original acquisition and equivalent to less than a millisecond). The missing data were then replaced through linear interpolation from the 5 preceding samples.

We then treated the LFP recordings as follows:

- The mean of the series was subtracted from the signal in order to bring all trials to a zero baseline.
- Boundary artifacts (arising from subject movement or instrumentation initialization) were removed following visual identification of their properties in the time series: they were typically 2–10 s in duration and seen as very high amplitude slow fluctuations in the signal. In order to avoid introducing differing lags between channels within a single trial, the signals were truncated at identical points such that all recordings were initialized at the same point in time (+5/-5 s from the start and end points).
- The data were down-sampled from the hardware's native sampling rate of 2400–200 Hz.
- All recordings were high-pass FIR filtered (applied to the data forward and reverse directions in order to prevent phase delays, order 300, passband at 4 Hz) to remove slow baseline fluctuations.

**Figure 2:**
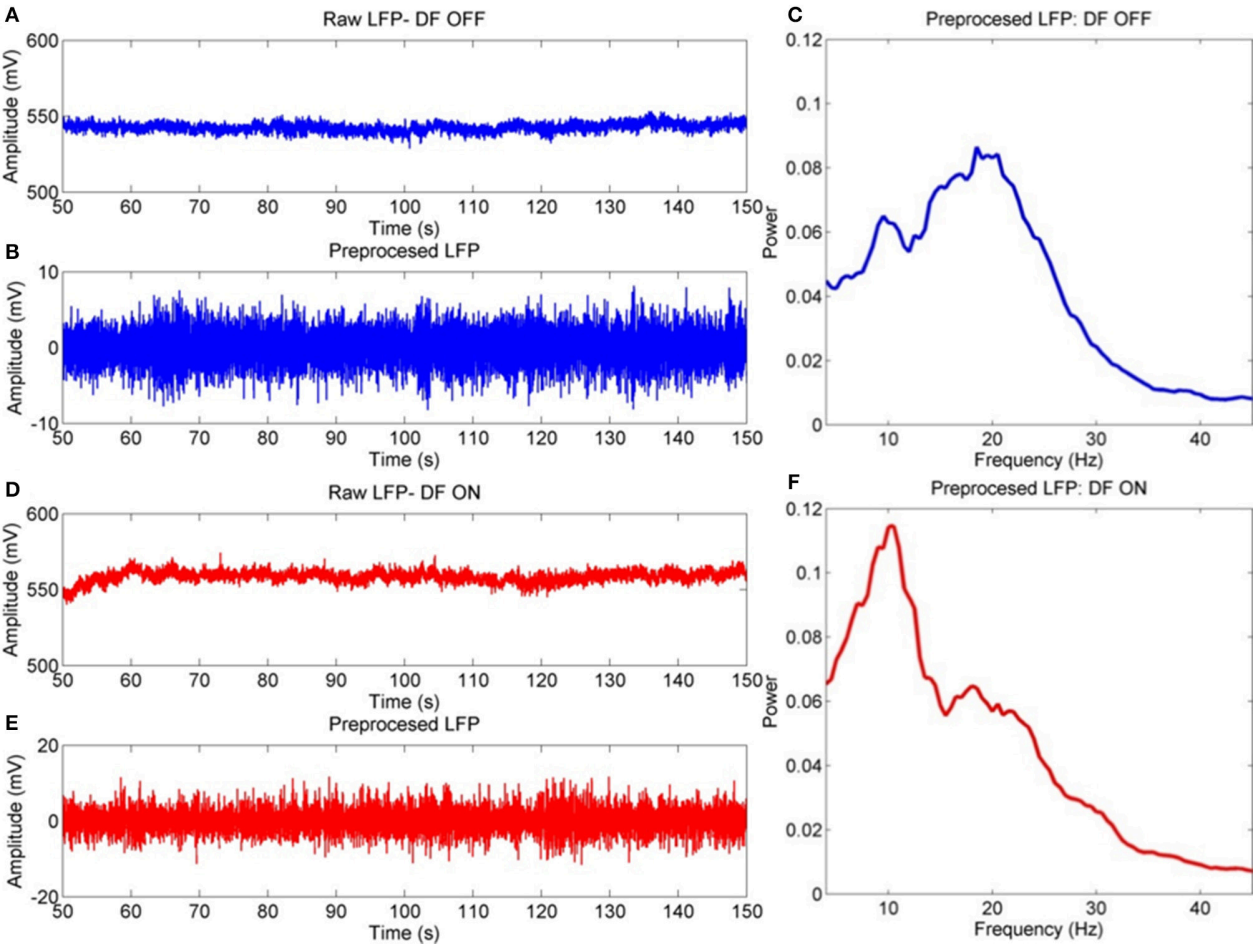
**Example procedure for LFP pre-processing**. Example signals are taken from the bipolar STNr R01 channel for a single subject. **(A)** Raw LFP contains artifacts such a high amplitude fluctuations (amplifier jumps), very low frequency baseline drifts, non-zero DC, and boundary artifacts at the extremities of the time series. **(B)** The pre-processed signal has been truncated, high pass filtered and large transients removed with missing data replaced via interpolation. The resulting spectra are in **(C)**, (**D–F**- Same as above but for the ON drug experiment). This data is used as it is for DFA-PS and cross correlation analyses. Spectral analyses are computed on epoched data in which bad trials are removed via Z-score thresholding.

Following pre-processing, signals had mean duration of 162.5 ± 6.7 s. For examples of the outcomes from pre-processing see Figure 2.

For all analyses that did not require continuous data (i.e., spectral analysis) we took further steps to reject artifacts by epoching the data into 2 s segments and then using Fieldtrip's artifact rejection routines (Z-score thresholding) to remove sections of data.

#### HPI pre-processing

In order to quantify subject movement during the recording, MEG HPI data was collected simultaneously to LFP recordings. HPI data were treated in the same way as the artifact-rejected LFP data. Signals were down sampled and large jump artifacts in signal were removed via Z score threshold followed by linear interpolation between gaps. The validity of HPI localization was verified by examining the pairwise distances between the fiducial coils which should remain constant through each trial (Oswal et al., 2015). All the data were found to be valid for this group of subjects.

#### Spectral analysis

Spectral estimates were made using Thomson's multi-taper method (Thomson, 1982) implemented in Fieldtrip. Multi-taper estimates were made from an average of 2 s long epochs using a set of orthogonal window functions to yield a 1.5 Hz frequency smoothing kernel (Prieto et al., 2007). When creating group average spectra, normalization was achieved by dividing the power spectra by its integral between 4 and 48 Hz.

Three predefined frequency regions of interest were defined at 5–12, 13–20, and 21–30 Hz, labeled alpha-theta, low and high beta ranges respectively as previously outlined in the literature (Oswal et al., 2013). These were then confirmed via visual identification of discrete peaks in the group mean power spectra. The power in a band was calculated as the integral of the power in the range of the frequency band of interest. For analysis of covariance of power with coherence we made the measures comparable by reporting power as an average between the two respective channels of interest as indicated in the pair configurations in Figure 1B.

#### Functional connectivity analysis: spectral coherence

The spectral coherence between two signals *X(t,n)* and *Y(t,n)* was first determined by estimation of the cross-spectral density, which encapsulates both the mean phase-difference between signals, as well as the correlation of power between *S*_*x*_*(*ω*)* and *S*_*y*_*(*ω*)*.

The cross-spectral density (CSD) is given by:

$$\begin{array}{l} S_{xy}\left(\omega ,n\right)=E\left[S_{x}\left(\omega ,n\right)S_{y}{\left(\omega ,n\right)}^{*}\right] \end{array}$$

where *S*_*x*_ is the complex-valued Fourier spectra, *E* is the expected value of a function and ^*^ indicates the complex conjugate. Multiplying by the complex conjugate effectively inverts the sign of the imaginary part of *S*_*y*_ such that multiplication of opposing imaginary exponents results in a subtraction. Peaks in the cross spectrum reveal frequencies that are common to both *x* and *y* as weighted by the degree of phase consistency and their respective amplitudes.

The magnitude squared coherence is the squared modulus of the CSD normalized by the respective power spectra of its constituents:

$$\begin{array}{l} Coh^{2}\left(\omega \right)=\frac{{\left|S_{xy}\left(\omega \right)\right|}^{2}}{S_{xx}\left(\omega \right)S_{yy}\left(\omega \right)} \end{array}$$

The coherence can be thought of as the spectral equivalent to the coefficient of determination *R*^2^. It is therefore a real number such that for any given frequency, 1 is equal to maximal correlation between two time series and 0 indicates an absence of correlation (Halliday et al., 1995). The 95% confidence limits for this measure can be calculated analytically (see below).

#### Non-zero phase lag functional connectivity analysis: WPLI

Coherence is sensitive to spurious effects resulting from volume conduction between the two signals of interest (Bastos and Schoffelen, 2016). These are assumed to occur at zero phase lag. In order to overcome this problem, several methods have been developed including analysis of the imaginary part of coherence (Nolte et al., 2004); and the phase lag index (PLI) (Stam et al., 2007). In this case we have opted for the WPLI as it has been demonstrated to be robust to simulated volume conduction effects, robust to noise, and sensitive to a range of relative phase distributions (Vinck et al., 2011). Like coherence WPLI is scaled 0–1.

Exact details of the WPLI method can be found in Vinck et al. (2011), but briefly WPLI is a modification of PLI which effectively quantifies non-zero distribution of phase differences by taking the expected value of the sign of the phase difference. By measuring the contribution of only the non-zero elements of the distribution of phase differences PLI provides an estimate of functional connectivity that is uncontaminated by zero phase lag interactions some of which may be due to volume conduction. WPLI is a modification of PLI that adjusts for bias by weighting the phase differences by the magnitude of its corresponding imaginary component of the cross spectrum.

### Non-linear characterization of synchronization dynamics

For linear measures of coherence to be significant there needs to be consistency over time of the phase and frequency of the two signals. It is possible to characterize phase interactions that are changing across time through an estimate of the long-range temporal correlation of the phase difference time series. Here we utilize Detrended Fluctuation Analysis for Phase Synchrony (DFA-PS; Botcharova et al., 2014). This method estimates the scaling statistics of the instantaneous rate of change of the phase-difference between two signals of interest.

#### Detrended fluctuation analysis (DFA)

A signal is said to exhibit long-range dependencies if non-zero correlations exists between its samples even when separated by long time intervals. In other words, its auto-correlation function has a slow decay. In the case that this decay may be approximated by a power law, DFA provides a way of estimating the extent of so-called “long range temporal correlations (LRTCs)” present in the signal (Peng et al., 1995; Shao et al., 2012). Estimation of the power law exponent α directly from the autocorrelation function is usually impractical due to noisy estimates at large lags as well as non-stationarity in the data. Instead we estimate the Hurst exponent *H* which is linearly related to the exponent α of the power law by α = 2 − 2*H*.

The DFA estimation is achieved by dividing the signal of interest into a number of equally spaced boxes in which the root mean square (RMS) deviation of the linearly detrended signal is computed. This process is repeated over a logarithmic range of box scales. The minimum scale at which this may be achieved is determined by the wavelength of the oscillation of interest. In order to obtain a good sample of oscillatory fluctuations we set the minimum box size at 6 times the wavelength of the lower boundary of the bandpass. The maximum is set such that detrended fluctuations are computed for at least 8 boxes. The mean RMS fluctuation for each box size is then plotted against box scale on a double logarithmic plot. If the resulting *fluctuation plot* is linear then the gradient of its least squares regression is the estimated Hurst exponent *H* (Peng et al., 1994, 1995).

The exponent H characterizes the extent of temporal dependencies in a signal. White noise i.e., a “memoryless” signal has an exponent *H* of 0.5 in which correlations in time rapidly decay, whilst exponent values >0.5 characterize signals that exhibit “memory” in the form of LRTCs. Increasing exponent size up to a value of 1.0 indicates more persistent temporal correlations and an increasing amount of order in the signal (Hardstone et al., 2012).

#### DFA for phase synchronization

As we are interested in phase dynamics we apply DFA to the derivative of the instantaneous phase difference between two signals as described in Botcharova et al. (2014). The pre-processed continuous signals *X*_*n*_*(t)*are first filtered with a 5 Hz wide passband centered at the frequency with peak coherence λ to yield the oscillations $\tilde{X_{n}}$ at the frequency band of interest. Filter orders were chosen to include three cycles of the slowest component frequency. This approach has been used in previously studies (e.g., Linkenkaer-Hansen et al., 2001; Botcharova et al., 2015). The band passed signal is given by:

$$\begin{array}{l} \tilde{X_{n}}=X_{n}\left(\lambda ,t\right) \end{array}$$

The analytic signal is then computed via the Hilbert transform:

$$\begin{array}{l} A_{n}e^{i\phi_{t}\left(n\right)}=H\left[\tilde{X_{n}}\right] \end{array}$$

and the instantaneous phase is calculated such that:

$$\begin{array}{l} \phi_{1}\left(t\right)-\phi_{2}\left(t\right)=\tan^{-1}\left\{H\left[\tilde{X_{1}}\right]-H\left[\tilde{X_{2}}\right]\right\} \end{array}$$

In order to use DFA, the series ϕ_1_(*t*)−ϕ_2_(*t*) must be converted to a bounded process. In order to do this we take the rate of change (approximate first derivative) of the time series of the phase difference:

$$\begin{array}{l} S\left(t^{-1}\right)=\frac{d\left(\phi_{1}\left(t\right)-\phi_{2}\left(t\right)\right)}{dt} \end{array}$$

This process is illustrated in Image 3 of the Supplemental Data. The signal represents the rate of change of phase difference between the two series. When S is at zero the signals have a constant phase relationship (Pikovsky et al., 2003). Previous work measuring the DFA of phase synchrony in noisy Kuramoto oscillator models has suggested that the scale free statistics of the rate of change of phase difference are increasingly persistent (as measured by an increasing α exponent) as the system approaches the phase transition leading to synchronization (Botcharova et al., 2014).

#### Assessing fluctuation plot linearity

At the onset of full synchronization between pairs of oscillators there is a collapse in the scaling and observed fluctuation plots become non-linear. From this observation we stress the importance of assessing plot validity in order to yield an interpretable exponent. In this analysis we remove exponents associated with non-linear DFA plots via a model comparison approach inspired by that previously reported in Botcharova et al. (2013). This method compares a range of potential underlying models that are fit to the fluctuation plot and discriminates between them using an estimate of the log model evidence (the free-energy approximation) using a defined level of stringency. This method makes use of the parametric empirical Bayes (PEB) estimator included in the SPM analysis package (Friston et al., 2002; Penny et al., 2011). The free energy is an approximation to the lower bound of the log model evidence and consists of an accuracy and complexity term:

$$\begin{array}{l} \log p\left(y|m\right)=Accuracy\left(m\right)\;-\;Complexity\left(m\right) \end{array}$$

Model fits are compared using an approximation of the Bayes factor *K* for comparison of models *i* and *j:*

$$\begin{array}{l} K_{ij}=\frac{p\left(y|m=i\right)}{p\left(y|m=j\right)} \end{array}$$

We make an approximation to *K* by taking the difference of the free energies between the model set outlined in Appendix I in the Supplementary Material (for more details see Penny et al., 2010). The best fitting model is assumed to be linear and only rejected when *-2 log(K)* > *4*, yielding “positive” evidence in favor of an alternative model over the linear model. It is important to emphasize that in contrast to the previously described methods, fluctuation plots are only rejected in the case that there is strong evidence in favor of an alternative model over the linear model. For examples of the validation process see Supplementary Image 1. In this method the level of stringency is defined by the aforementioned criterion and set to match levels of evidence agreed in the established literature (Kass and Raftery, 1995).

#### Permutation statistics

In order to determine whether the observed exponents for phase ordering were significantly different from those arising from random fluctuations we computed a permutation statistic. For each set of exponents (all frequency bands, ON and OFF L-DOPA, inter- and intra-nuclear) we computed a null-distribution, shuffling the time derivative of the phase difference time series and computing a set of 1000 DFA-PS exponents. This technique preserves the spectral power in the signal but randomizes the temporal structure of the phase difference. *P*-values were then estimated using:

$$\begin{array}{l} P=\frac{b+1}{m+1} \end{array}$$

where *b* is the number of DFA-PS exponents computed from the randomized series that exceed the mean exponent observed in the actual data, and *m* is the number of permutations used (*m* = 1000) (Phipson and Smyth, 2010). A *P*-value was determined with the null hypothesis that the mean of the observed exponents was not sufficiently different from that of those computed from a series with uncorrelated phase dynamics.

### Subdivision of STN channels

In order to avoid introducing selective bias to our results we avoided the presumption that the channel with highest beta power is closest to STN, as has been done in previous studies. Since part of our analysis is concerned with functional connectivity, we did not wish to reduce the potential dimensions for cross channel coherences nor make assumptions regarding the exact positioning of contacts. Channels were created from a bipolar montage of contacts as shown in Figure 1A.

Channel pairings were divided into two groups: pairs of channels on the same electrode are presumed to be *intra-nuclear*- that is within the region of one STN. Pairings of channels between left and right STN electrodes are termed *inter-nuclear*. Altogether there are 6 possible intra-nuclear combinations (3 for each nucleus left and right) and 9 left-right inter-nuclear combinations (see Figure 1B). When computing summary statistics for bivariate analyses (i.e., coherence) results were reported as an average metric for all pairs in the group. For intra-nuclear pairs the group was split into two averages for pairs originating from either the left or the right STNr. Inter-nuclear results are reported as one average of all 9 pairs. In some cases univariate metrics (power) were entered into regressions alongside the bivariate measure, when this was done the average power of the two channels in each pair was considered.

### Statistical testing

For further details concerning reporting of statistics, testing, outliers and computation of a general linear mixed model (GLMM) please refer to Appendix II in Supplementary Material.

## Results

### Electrode localization

From the total of 96 contacts (12 ^*^ 4 ^*^ 2), 26 (27%) were outside the STN, 19 (20%) were at the border of the STN and 51 (53%) were inside the STN. For 23/24 hemispheres one or more contact points were inside or at the border of the STN.

### Spectral analysis

#### Dopaminergic modulation of band power and frequency

**Figure 3:**
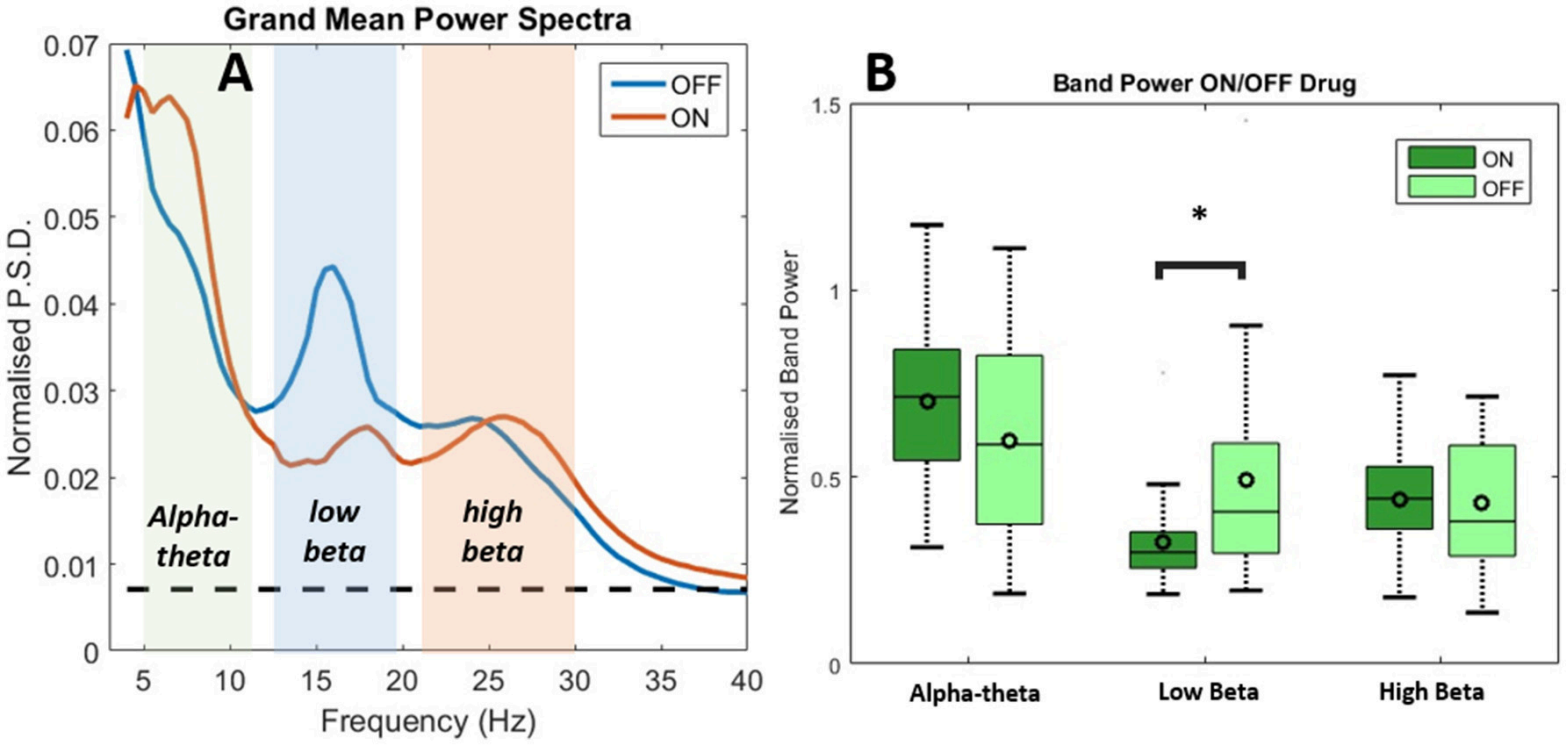
**Analysis of power spectra and statistics for LFP recordings from STNr when either ON or OFF L-DOPA**. **(A)** Group level power spectra for all 12 subjects and for all channels. The dashed line indicates the 95% analytic confidence limit. There is a clear L-DOPA associated modulation in the alpha-theta band (5–12 Hz) and low beta band (13–20 Hz). There is also significant power in the high beta band (21–30 Hz) although L-DOPA modulation is less prominent. **(B)** Comparison of band power ON and OFF L-DOPA indicated an increase in alpha-theta power in the ON state whilst low beta was shifted in the OFF. There were no significant changes in mean power detected for high beta. The effect of OFF drug modulation in the low beta band was statistically significant as it was below the Bonferonni corrected P-value. Significance stars indicate range of *P*-values for a repeated measures ANOVA (^*^P < 0.05).

Results of spectral analysis can be seen in Figure 3 and Table 2. Visual inspection of the spectra as well as analysis of peak frequencies confirms the suitability of the selected bands of interest (alpha-theta: 5–12 Hz, low beta: 13–20 Hz, and high beta: 21–30 Hz). Individual patient's spectra had varying profiles with some showing more pronounced alpha-theta or beta band peaks. In pooled spectra there were three peaks at frequencies 7.24 ± 1.55, 16.03 ± 2.30, and 25.84 ± 2.56 Hz (see Figure 3A) were apparent. The spectral peaks fall in the middle of pre-defined bands suggesting that peaks were not split when segregating bands to compute total power. No significant shifts in frequency were determined for any of the three bands when comparing recordings ON and OFF drug (see Table 2 for results of testing).

**Table 2:** **Table of results for group level spectral analysis of STN data and comparison of ON/OFF drug effects**.

	**ON**		**OFF**				**ANOVA**
	**Mean**	**SD**	**Mean**	**SD**	**ON-OFF**	**d.f**.	***P*-Value**
**PEAK FREQUENCY (HZ)**							
Alpha-theta	7.236	1.554	8.222	2.328	−0.986	23	0.242^†^
Low beta	16.028	2.302	16.319	2.308	−0.292	23	0.852^†^
High beta	25.840	2.557	24.819	2.660	1.021	23	0.090
**SPECTRAL POWER IN BAND**							
Alpha-theta	0.702	0.236	0.595	0.283	0.106	23	0.041
Low beta	0.327	0.125	0.494	0.284	−0.167	23	**0.012**^†^
High beta	0.441	0.144	0.433	0.172	0.009	23	0.822

Mean and standard deviations of band power and peak frequency in the Alpha-theta, low beta, and high beta ranges are shown as averages across all channels. P-values are reported for repeated measures ANOVA in which the significance of the differences in mean values ON and OFF drugs are tested (P < 0.05),

† indicates cases where non-parametric statistics were used due to non-normality in the sample. Results in bold indicate P-values that remain below the Bonferroni corrected decision level termed α^*^ (see section “Reporting of Statistics” in supplemental material). The number of tests considered is equal to total number conducted for each method [i.e., Frequency − 3 tests (1 ANOVA × 3 Bands)].

Analysis of the relative power in bands showed a significant change in average power following medication (Figure 3B and Table 2) for alpha-theta and low beta but not for high beta. Low beta band power is increased in patients following withdrawal of L-DOPA with a difference in mean (normalized) power between ON and OFF states of −0.17±0.28 (repeated measures ANOVA(23), *P* < 0.05) which is in good agreement with previously published findings (Priori et al., 2004; Weinberger et al., 2006; Hammond et al., 2007). There was no significant ON vs. OFF modulation in the high beta band.

At approximately 8 Hz there is a clear peak in both the subject and group level power spectra. Activity at this frequency band will be termed alpha-theta although previous studies looking at similar frequency ranges have used terms such as *slow oscillatory activity* (Alonso-Frech et al., 2006) or *sub-beta* (Kato et al., 2015). Similar to previous studies we have found that alpha-theta power is raised in the ON drug state. We demonstrate a significant increase of 0.106 ± 0.26 in mean normalized alpha-theta power for all channels when pooled (repeated measures ANOVA (23), *P* < 0.05) although this result did not remain when correcting for multiple comparisons.

#### Correlation of bandpower with OFF state UPDRS and changes in UPDRS associated with L-DOPA treatment

**Figure 4:**
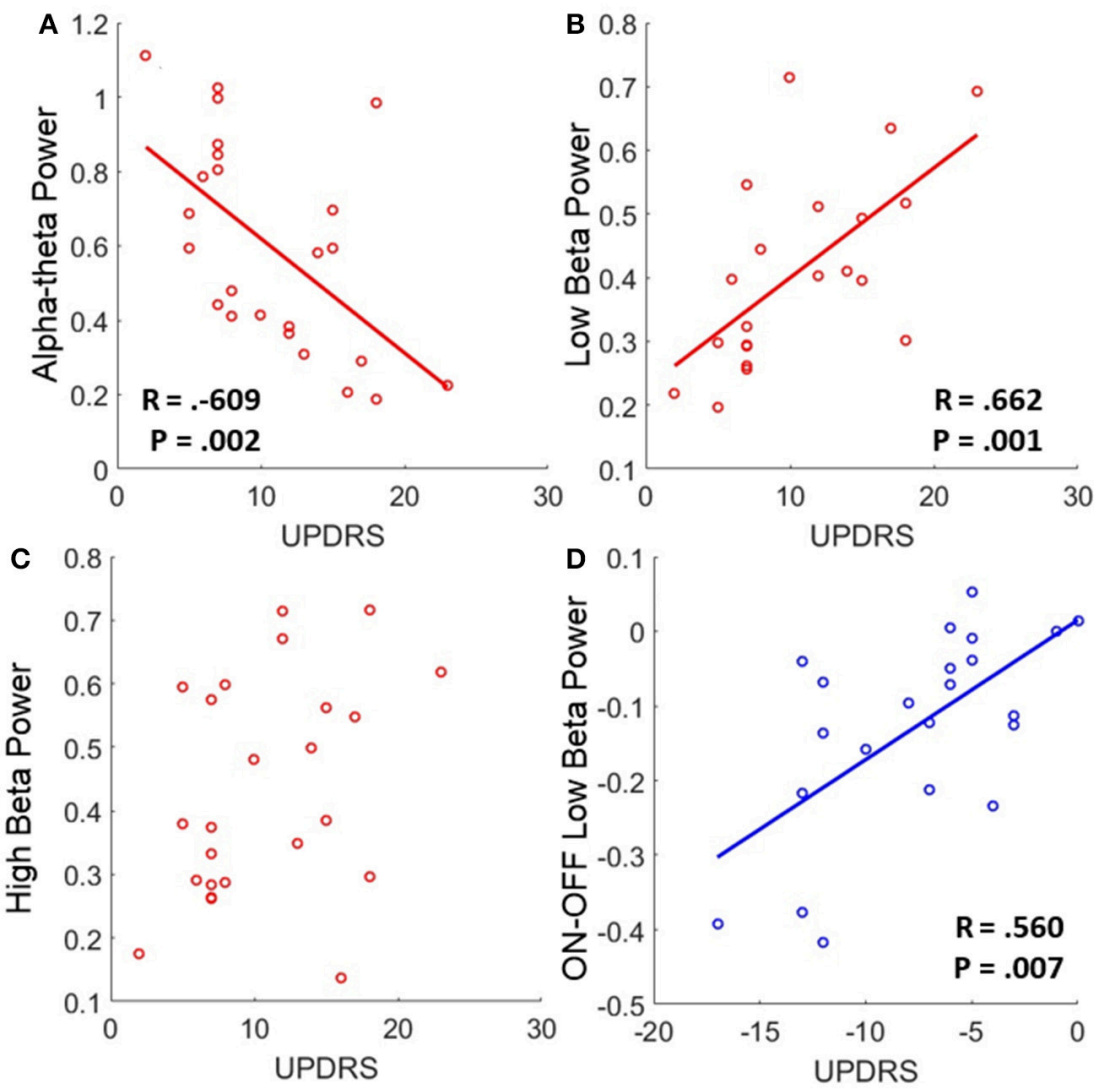
**Correlations of band power with UPDRS for bradykinesia/rigidity scores**. **(A–C)** Scatter plots of band power for alpha-theta, low beta, and high beta bands with OFF state UPDRS. Correlations were first determined to be significant using Spearman's test and in the case of significance a linear regression was plot. The corresponding R is reported alongside as well as the *P*-value for the test. Low alpha-theta power was associated with less severe motor symptoms whilst for low beta the opposite was true. No relationship was found for high beta band. **(D)** Correlation of ON-OFF low beta band power with ON-OFF UPDRS. The positive correlation was found to be significant and the subsequent linear regression is shown. The relationship suggests that larger reductions in low beta power when ON drug are associated with a greater therapeutic reduction in motor symptoms when ON compared to OFF.

Low beta band power is positively correlated with clinical scores in the OFF state [*r*_(19)_ = 0.662, OFF; see Figure 4B, **Table 5**] and when entered into a GLMM (**Table 7**) it was able to account for a third of the UPDRS variance (*P* = 0.001, Ω^2^ = 0.343). In order to determine if this effect was related to clinical improvement with L-DOPA treatment we correlated the ON minus OFF beta power change with the ON minus OFF difference in UPDRS where we found a significant linear effect [*r*_(20)_ = 0.560, ON-OFF; see Figure 4D]. In a GLMM this accounted for 39% of the variance [*P* = 0.002, Ω^2^ = 0.386, ON-OFF]. This result suggests that the larger the OFF to ON state reductions in beta power are the larger the decrease in the severity of clinical motor symptoms when treated with L-DOPA. No statistically significant correlations were found in high beta range (see Figure 4C).

There is an opposite effect for power in the alpha-theta band which correlates negatively with OFF drug UPDRS [*r*_(22)_ = −0.609, OFF], suggesting that increased alpha-theta power is associated with less severe clinical disease states (see Figure 4A). When entered into the mixed model we found that alpha power could account for a similar degree of explained variance (*P* = 0.002, Ω^2^ = 0.331). We found no significant correlation for alpha-theta power with drug-induced clinical improvement.

In order to assess the predictive power of both alpha-theta and low beta power together we entered them into a multiple regression. However, neither alpha-theta nor low beta power were identified as significant predictors of OFF state UPDRS (see **Table 7**) when entered together. This effect arises due to collinearity between the two regressors that significantly inflates error estimation. Because there is a strong negative correlation between alpha and low beta band it is not possible to determine which the strongest predictor is.

### Analysis of functional connectivity

#### Dopaminergic modulation of coherence

**Figure 5:**
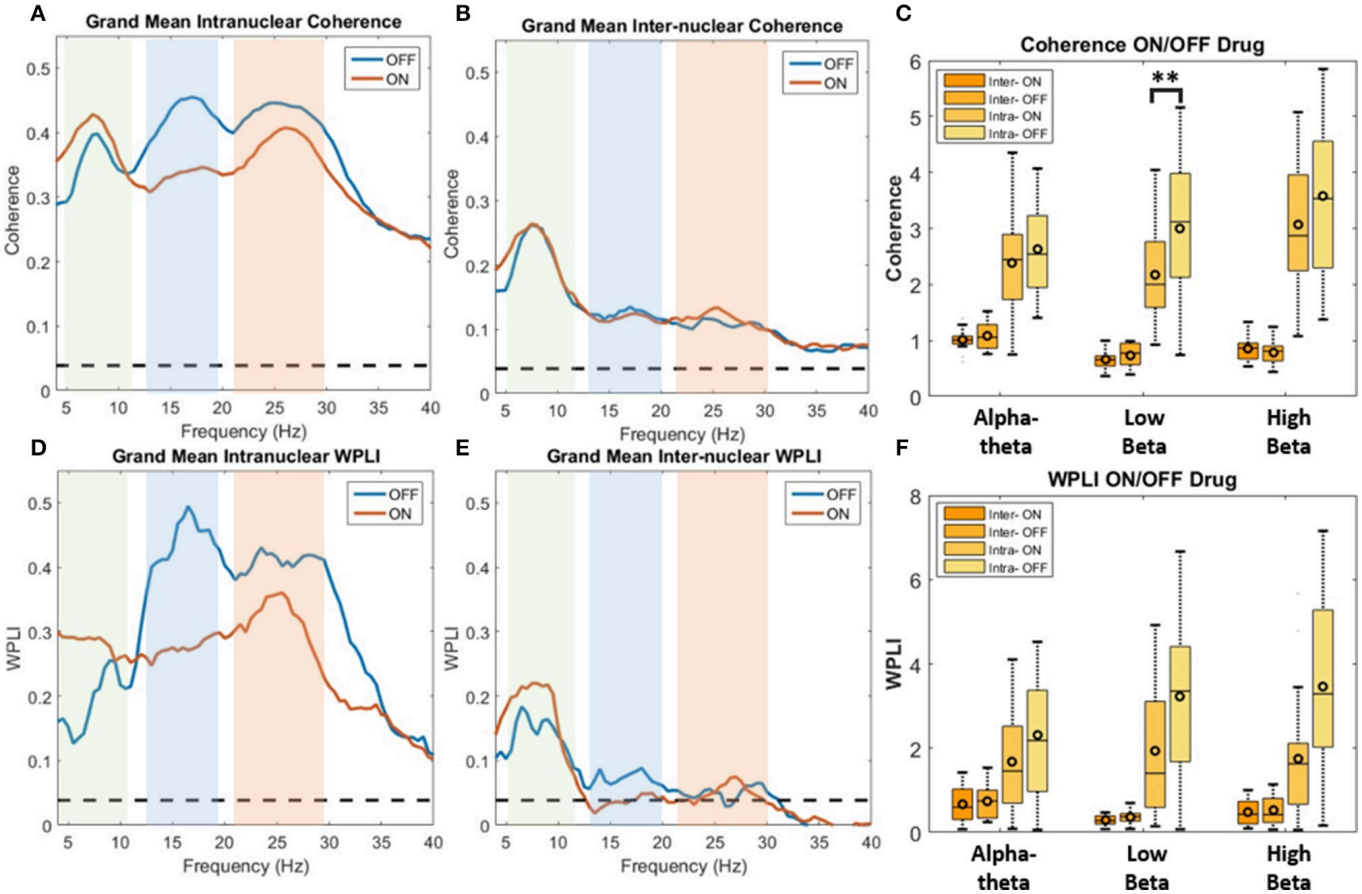
**Analysis of STNr functional connectivity within nuclei (intra-nuclear) and between left and right nuclei (inter-nuclear) using coherence (top row) or weighted phase lag index (bottom row)**. All results are presented as pooled data across all 12 subjects ON and OFF L-DOPA. Dashed line on spectra represent the analytic 0.95% confidence limit. Significance stars for boxplots indicate range of *P*-values for a repeated measures ANOVA (^**^P < 0.01). **(A)** Pooled intra-nuclear coherence spectra. Plots show strong coherence (>0.3) across the three bands of interest. **(B)** Inter-nuclear coherence spectra. Although weaker in comparison to the intra-nuclear coherence, bilateral STNr coherence remains above significance level for the full frequency bands analyzed. Most however is focused in the alpha-theta band. **(C)** Boxplot comparison of AUC coherence when ON and OFF L-DOPA. There is evidence for an increase in low and high beta coherence when OFF drug. No significant modulations were found for the inter-nuclear comparisons. **(D)** Pooled intra-nuclear WPLI spectra. There still remains significant coherence within STNr when removing the zero-lag component. **(E)** WPLI inter-nuclear spectra. All frequencies show attenuation in comparison to **(B)** although there is still significant alpha-theta band connectivity. **(F)** Boxplot comparison of AUC WPLI when ON and OFF L-DOPA. The up-modulation of low beta band connectivity within nuclei in response to L-DOPA when correcting for potential volume conduction effects did not meet the Bonferroni corrected significance threshold.

In order to determine if treatment with dopaminergic medication alters functional connectivity within STNr (*intra*-nuclear) or bilaterally between STNr (*inter*-nuclear) we measured the coherence between channels. The pooled coherence spectra are shown (Figures 5A–C, top row) with summary statistics reported in Table 3. In the pooled intra-nuclear coherence spectra there are peaks at ~8 Hz; ~16 Hz; and another at ~28 Hz. These peaks could be clearly observed in coherence estimates from individual subjects. Statistics for differences in the mean values of coherence are reported as an area under the curve measure (AUC, termed here *integrated coherence*). Comparisons between ON and OFF conditions suggest that the effect is significant within the low beta band with intra-nuclear coherence exhibiting a decrease of −0.76±0.57 (repeated measures ANOVA (21) *P* < 0.01, *intra*-) when the patient is ON drugs, suggesting that L-DOPA reduces beta frequency intra-nuclear synchronization. There was also a comparable yet weaker effect in the high beta band (−0.416±0.564, repeated measures ANOVA (22) *P* < 0.05) but this did not survive Bonferroni correction.

**Table 3:** **Table of results for group level coherence analysis and comparison of ON/OFF drug effects**.

	**ON**		**OFF**				**ANOVA**
	**Mean**	**SD**	**Mean**	**SD**	**ON-OFF**	**d.f**.	***P*-value**
**ON/OFF L-DOPA COMPARISON OF AUC COHERENCE**							
**Alpha-theta**							
Intra	2.376	0.922	2.635	0.777	−0.259	23	0.214
Inter	1.006	0.240	1.047	0.228	−0.040	10	0.600
**Low beta**							
Intra	2.165	0.952	2.927	1.335	−0.762	21	**0.002**
Inter	0.656	0.187	0.712	0.222	−0.056	10	0.330
**High beta**							
Intra	3.079	1.205	3.495	1.289	−0.416	22	0.036
Inter	0.846	0.242	0.784	0.221	0.061	11	0.403
**ON/OFF L-DOPA COMPARISON OF AUC WPLI**							
**Alpha-theta**							
Intra	1.670	1.199	2.219	1.375	−0.548	23	0.083
Inter	0.661	0.440	0.736	0.440	−0.074	12	0.520
**Low beta**							
Intra	1.935	1.593	3.151	1.924	−1.215	23	0.041^†^
Inter	0.280	0.146	0.340	0.154	−0.061	10	0.230
**High beta**							
Intra	1.746	1.528	3.137	2.089	−1.390	21	0.014^†^
Inter	0.480	0.303	0.523	0.379	−0.043	12	0.724

Mean and standard deviations of the integrated coherence and WPLI coherence in the Alpha-theta, low beta, and high beta ranges are shown for intra-/inter- nuclear and all pairs of contacts. Outlier have been removed. Results from repeated measures ANOVA to examine the significance of drug related modulation in the mean value of coherence are shown in the final column (P < 0.05),

† indicates cases where non-parametric statistics were used due to non-normality in the sample. Results in bold indicate P-values that remain below the Bonferroni corrected decision level termed α^*^ (see section “Reporting of Statistics” in supplemental material). The number of tests considered is equal to total number conducted for each method [i.e., Coherence − 6 tests (1 ANOVA × 3 Bands × 2 Pairings)].

For inter hemispheric functional connectivity, overall coherence is lower when compared to that observed for intra-nuclear STNr recordings (see Figure 5B) but it remained above the 95% confidence interval in the range 4–40 Hz. There is a well-defined peak coherence in the alpha-theta band with a maximum coherence at ~0.25. No statistically significant dopaminergic modulation of bilateral functional connectivity was observed (see Table 3).

#### Dopaminergic modulation of weighted phase lag index (WPLI)

In order correct for potential volume conduction effects on the coherence measure we used the WPLI estimate of functional connectivity which is robust to zero phase-lag correlations (Vinck et al., 2011). The results of the pooled WPLI are qualitatively similar to those of standard coherence (see Figures 5D–F, bottom row).

WPLI statistics are shown in Table 3 and summarized in the boxplots in Figure 5F. For intra-nuclear pairs we observed that the overall coherence is reduced when comparing WPLI to standard coherence (compare Figures 5A,D). The effect size of L-DOPA on low beta band connectivity was more evident when analysis was conducted with WPLI yielding an ON-OFF difference of −1.22±0.67 (repeated measures ANOVA (23) *P* < 0.05, *intra*); from this we conclude that standard coherence and WPLI indicate that L-DOPA reduces beta frequency synchronization within the STNr in Parkinson's patients.

For bilateral inter nuclear STNr connectivity, correction for zero lag interactions heavily attenuated coherences at frequencies >15 Hz to levels to below or close to the significance threshold (see Figure 5E). The alpha-theta band interaction observed when using coherence remained in the WPLI although was also attenuated. The overall reduction in coherence would suggest that zero-lag interactions are present in the inter-hemispheric connectivity between STNr. These effects cannot be explained on the grounds of removal of volume conduction and they may reflect the fact that coherence is detecting a zero lag common input to left and right STNr which has been removed by the WPLI method. No statistically significant modulatory effect of L-DOPA was observed for left to right STNr connectivity as measured with WPLI (see Table 3).

#### Correlation of connectivity measures with OFF state motor symptom severity

Significant correlations of within nucleus coherence with OFF state UPDRS were found for all three bands although only low beta band interactions survived when removing zero-lag coupling using the WPLI metric (Table 5) as well as when correcting for multiple comparisons. Intra-nuclear STNr coherence in the low beta band was found to positively correlate with UPDRS [*r*_(22)_ = 0.643, OFF i*ntra*-] suggesting that UPDRS symptom severity increases with increased low beta band coherence. There was also an OFF state UPDRS correlation found for high beta coherence [*r*_(22)_ = 0.460,OFF *intra*-] although was not detectable using the WPLI measure and the effect was not independent of power in any of the three bands.

The effect for low beta remained when measuring connectivity using WPLI [*r*_(22)_ = 0.575,OFF *intra*-] and was demonstrated to be a significant predictor when entered into a GLMM (*P* < 0.001, Ω^2^ = 0.41). In order to determine if this effect was separate to that of low beta power, the two measures were entered as covariates into a multiple regression. It was found that only power was a significant regressor in the model suggesting that any variance explained by WPLI is contained within that explained by power. Thus whilst linear measures of functional low beta band intra-nuclear connectivity related to the severity of the motor OFF state PD, these effects cannot be separated from those of power in the same frequency band.

There was no effect found when correlating beta coherence or WPLI change ON minus OFF with clinical improvement following L-DOPA. No significant OFF UPDRS correlations were found for inter-nuclear coherences.

### Measuring phase ordering in STN interactions

#### Dopaminergic modulation of DFA-PS exponents

We applied DFA-PS to the LFP data at frequency bands centered at the peak of the spectral coherence for each respective band. A 2.5 Hz half-band width filter was chosen to allow for changes of the coherent frequency over time, covering the full width of the coherent band, as well as to avoid removing phase dynamics that could be missed with too tight a passband (Boashash, 1992). When reporting exponents, the validation technique described in Section Assessing Fluctuation Plot Linearity was utilized to identify and remove exponents originating from non-linear DFA plots. For examples of the outcomes of the validation procedure see Image 1 of the Supplementary Material.

**Figure 6:**
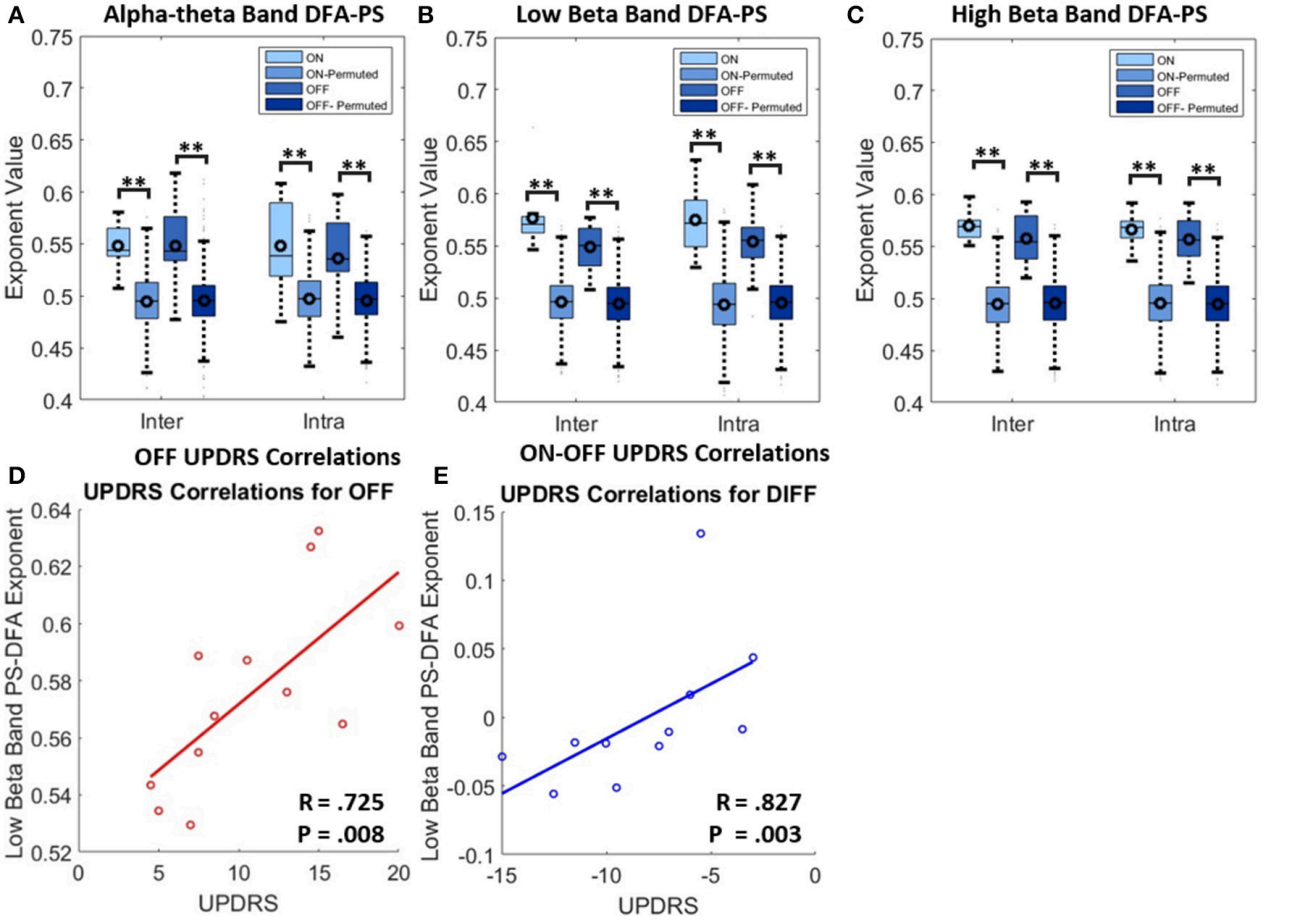
**Results for analysis with PS-DFA measure for phase ordering between intra-nuclear and inter-nuclear signal pairs**. **(A–C)** Boxplots for PS-DFA exponent values. Comparisons are made between exponents computed from the recorded signals and from a series of 1000 permutated signals. Significant differences were computed using the permutation statistics described in the text *P*-values (^**^P < 0.01). No significant changes in exponents were found with application of L-DOPA although all were significant different from signals without any phase ordering. **(D)** Low beta band bilateral PS-DFA exponents are positively correlated with worse motor symptoms. **(E)** ON-OFF difference correlations. The correlations suggest that patients with a larger decrease in exponent ON drug also show the best therapeutic effect.

Results from DFA-PS analysis demonstrate that the majority of fluctuation plots are linear and have non-trivial exponents when compared to exponents from shuffled data (i.e., no correlations present, exponent ~0.5) with *P*-values (computed as in Section Permutation Statistics) for all comparisons demonstrating that exponents were significantly different from those determined from purely random phase signals (see Table 4 and Figures 6A–C). As the majority of fluctuation plots in the alpha and low beta bands were validated as linear, there is evidence for the existence of temporal persistence in the phase relations of the signals of interest indicating long-range temporal correlations (LRTCs). i.e., temporal order within the fluctuation rates of change of phase difference (See Botcharova et al., 2014). Exponents values in the alpha-theta (0.55 ± 0.04, ON, *intra*-) and low beta (0.58 ± 0.03, *ON intra*-) bands are in the range that is in agreement with those previously reported in the motor cortex during a movement task (Botcharova et al., 2015).

**Table 4:** **Table of comparisons of PS-DFA Exponent values for ON or OFF levodopa**.

	**ON**			**OFF**			**Comparison**		**Total rejection rate**		
	**Mean**	**Std**.	**Perm. *P*-value**	**Mean**	**Std**.	**Perm. *P*-value**	**Difference**	***P*-value**	**ON**	**OFF**	**Difference**
**ON/OFF L-DOPA COMPARISON OF DFA-PS EXPONENTS**											
**Alpha-theta**											
Intra	0.548	0.021	$\underline{\underline{0.001}}$	0.548	0.044	$\underline{\underline{0.002}}$	0.000	0.980	0.031	0.073	−0.042
Inter	0.548	0.035	$\underline{\underline{0.001}}$	0.536	0.048	$\underline{\underline{0.001}}$	0.012	0.325	0.094	0.135	−0.042
**Low beta**											
Intra	0.577	0.031	$\underline{\underline{0.001}}$	0.575	0.033	$\underline{\underline{0.003}}$	0.001	0.929	0.458	0.302	0.156
Inter	0.549	0.023	$\underline{\underline{0.001}}$	0.554	0.029	$\underline{\underline{0.001}}$	−0.005	0.536	0.365	0.333	0.031
**High beta**											
Intra	0.570	0.014	$\underline{\underline{0.001}}$	0.566	0.016	$\underline{\underline{0.003}}$	0.003	0.626	0.490	0.490	0.000
Inter	0.558	0.022	$\underline{\underline{0.001}}$	0.557	0.021	$\underline{\underline{0.001}}$	0.001	0.901	0.417	0.406	0.010

Table shows the mean and standard deviation for exponent values for both ON and OFF L-DOPA treatment. Results for permutation testing of exponents against a set of exponents computed for phase shuffled data are also shown (see section Permutation Statistics). Significance for difference in means between ON and OFF conditions was tested using repeated measures ANOVA ($\underline{\underline{\text{P < 0.01}}}$). Exponents are validated using the PEB technique described in section Assessing Fluctuation Plot Linearity. The rejection rate is shown in the three right-most columns. Rejection rates are reported as the percentage of exponents removed from the total set that was computed. Results in bold indicate P values that remain below the Bonferroni corrected decision level termed α^*^ (see section “Reporting of Statistics” in supplemental material). The number of tests considered is equal to total number conducted for each method [i.e., DFA-PS Permutation Stats. − 12 tests (1 Permutation Statistic × 3 Bands × 2 Pairings × 2 Conditions)].

**Table 5:** **Bivariate correlations of UPDRS with power, coherence, and WPLI**.

	**OFF UPDRS**				**ON-OFF UPDRS Difference**			
	***r***	***R*^2^**	***P*-value**	**d.f**.	***r***	***R*^2^**	***P*-value**	**d.f**.
**BAND POWER CORRELATIONS WITH CLINICAL SCORES**								
**Alpha-theta**								
Intra	−0.609	0.331	$\underline{\underline{0.002}}$	22	−0.193	0.076	0.388	20
Inter	−0.616	0.386	0.033	10	−0.248	0.100	0.492	8
**Low beta**								
Intra	0.662	0.390	$\underline{\underline{0.001}}$	19	0.560	0.398	$\underline{\underline{0.007}}$	20
Inter	0.688	0.240	0.019	9	0.500	0.238	0.121	9
**High beta**								
Intra	0.365	0.133	0.080	22	0.267	0.053	0.242	19
Inter	0.518	0.229	0.084	10	0.200	0.003	0.558	9
**COHERENCE AUC CORRELATIONS WITH CLINICAL SCORES**								
**Alpha-theta**								
Intra	0.419	0.099	0.042	22	0.368	0.100	0.076	22
Inter	0.210	0.071	0.536	9	−0.510	0.431	0.094	10
**Low beta**								
Intra	0.643	0.409	$\underline{\underline{0.001}}$	22	0.396	0.092	0.062	21
Inter	0.415	0.232	0.205	9	0.133	0.061	0.683	10
**High beta**								
Intra	0.459	0.211	0.024	22	0.385	0.108	0.063	22
Inter	0.551	0.094	0.079	9	−0.503	0.343	0.099	10
**WPLI AUC CORRELATIONS WITH CLINICAL SCORES**								
**Alpha-theta**								
Intra	0.402	0.084	0.051	22	0.433	0.017	0.035	22
Inter	0.375	0.374	0.230	10	0.392	0.197	0.210	10
**Low beta**								
Intra	0.575	0.306	$\underline{\underline{0.003}}$	22	0.272	0.105	0.198	22
Inter	0.342	0.134	0.304	9	0.133	0.078	0.744	7
**High beta**								
Intra	0.074	0.007	0.732	22	−0.039	0.014	0.858	22
Inter	0.109	0.001	0.737	10	−0.636	0.346	0.030	10

Results are shown for OFF L-DOPA UPDRS and the DIFFERENCE (ON-OFF) in UPDRS (bradykinesia/rigidity). Correlations for each metric are computed for each band and for intra- and inter-nuclear sets. In the case of power, the inter-nuclear statistic is the average value between left and right nuclei. The Pearson correlation coefficient (r) is shown alongside the coefficient of determination (R^2^) for the corresponding linear regression. The P-value and the degrees of freedom for the Spearman's correlations are shown (P > 0.05; $\underline{\underline{\text{P > 0.01}}}$). Results in bold indicate P values that remain below the Bonferroni corrected decision level termed α^*^ (see section “Reporting of Statistics” in supplemental material). The number of tests considered is equal to total number conducted for each method [i.e., Power OFF UPDRS Correlation. − 6 tests (1 t-statistic × 3 Bands × 2 Pairings)].

Exponents obtained for LFP phase differences ON and OFF L-DOPA estimated for inter- and intra-nuclear pairings were compared (repeated measures ANOVA, see Figures 6A–C and Table 4). There were no significant differences found for mean exponents between ON and OFF drug suggesting that scaling statistics of phase interactions are not modulated by L-DOPA uniformly between patients. In the alpha-theta band most of plots were validated (>86%). The rate of rejection increased for both high and low beta bands where 55–70% of plots were linear and therefore found to be indicative of long range dependence.

#### DFA for phase synchrony and OFF drug clinical state

No significant correlation was found between OFF UPDRS and the exponent values obtained from analysis of STNr intra nuclear DFA-PS. Analysis of OFF state UPDRS with DFA-PS results identified positive correlations for exponent value for inter-nuclear pairs in the low beta frequency band [*r*_(10)_ = 0.725,OFF *inter*-] (see Table 6 and Figure 6D). These results demonstrate that increased order in the rate of change of phase differences of bilateral STNr LFP interactions correlates with increasing severity of bradykinetic symptoms in PD. When entered into a GLMM we demonstrated that low beta inter-nuclear DFA-PS exponents were significant independent predictors of OFF state UPDRS from that of that explained by differences in low beta power (*P* < 0.05, Ω^2^ = 0.472) (see Table 7). The effect for low beta DFA-PS was not demonstrated to be independent of alpha-theta power as a result of collinearity between the two variables. This correlation was shown to be significant [*r*_(10)_ = −0.685, *P* < 0.05,OFF *inter*-] and thus it was not possible to determine whether PS-DFA in the low beta band added explanatory power of OFF state UPDRS.

**Table 6:** **Bivariate correlations of UPDRS with PS-DFA Exponets**.

	**OFF UPDRS**				**ON-OFF UPDRS Difference**			
	***r***	***R*2**	***P***	**d.f**.	***r***	***R*2**	***P***	**d.f**.
**UPDRS CORRELATIONS WITH EXPONENTS FROM DFA FOR PHASE SYNCHRONY**								
**Alpha-theta**								
Intra	0.054	0.014	0.807	21	0.081	0.013	0.713	21
Inter	0.025	0.007	0.940	10	0.517	0.305	0.089	10
**Low beta**								
Intra	0.043	0.040	0.854	19	−0.197	0.097	0.450	15
Inter	0.725	0.471	$\underline{\underline{0.008}}$	10	0.827	0.328	$\underline{\underline{0.003}}$	9
**High beta**								
Intra	−0.204	0.041	0.402	17	−0.279	0.052	0.295	14
Inter	−0.074	0.013	0.820	10	0.355	0.109	0.286	9

Results are shown for OFF L-DOPA UPDRS and the DIFFERENCE (ON-OFF) in UPDRS (bradykinesia/rigidity). Exponents are validated using the PEB method described in Section 2.3.3. The Spearman correlation coefficient (r) is shown alongside the coefficient of determination (R^2^) for the corresponding regression. The P-value and the degrees of freedom for the correlations are shown ($\underline{\underline{\text{P > 0.01}}}$). Results in bold indicate P-values that remain below the Bonferroni corrected decision level termed α^*^ (see section “Reporting of Statistics” in supplemental material). The number of tests considered is equal to total number conducted for each method [i.e., DFA OFF UPDRS Correlation. − 6 tests (1 t-statistic × 3 Bands × 2 Pairings)].

**Table 7:** **GLM Regression Analysis**.

	**Bivariate Model**		**Alpha-theta Power**		**Low Beta Power**		**High Beta Power**	
	***P***	**Ω^2^**	***P***	**Ω^2^**	***P***	**Ω^2^**	***P***	**Ω^2^**
**UPDRS GLM REGRESSION ANALYSES**								
**Power- Intra-nuclear**								
Alpha-theta power	$\underline{\underline{0.002}}$	0.331			0.493	0.358	0.013	0.333
Low beta power	$\underline{\underline{0.001}}$	0.343	0.119	0.358			0.024	0.368
High beta power	0.067	0.133						
**Power- Inter-nuclear**								
Alpha-theta power	0.018	0.386			0.197	0.326	0.101	0.389
Low beta power	0.089	0.210						
High beta power	0.084	0.229						
**Coherence Intra-nuclear**								
Alpha-theta coherence	0.118	0.098						
Low beta coherence	$\underline{\underline{0.000}}$	0.409	0.032	0.450	0.059	0.448	$\underline{\underline{0.002}}$	0.418
High beta coherence	0.018	0.211	0.585	0.339	0.407	0.364	0.073	0.244
**Coherence Inter-nuclear**								
Alpha-theta coherence	0.378	0.039						
Low beta coherence	0.095	0.205						
High beta coherence	0.309	0.061						
**DFA-PS Intra-nuclear**								
Alpha-theta exponent	0.574	0.023						
Low beta exponent	0.359	0.181						
High beta exponent	0.380	0.028						
**DFA-PS Inter-nuclear**								
Alpha-theta exponent	0.776	0.007						
Low beta exponent	$\underline{\underline{0.007}}$	0.471	0.100	0.514	0.039	0.472	0.017	0.529
High beta exponent	0.700	0.013						

Results from statistical modeling of UPDRS regression with spectral features such as power in band, coherence, and PS-DFA exponents. The bivariate regressions are shown in the first column with the respective P-value of the predictor for UPDRS and the overall Ω^2^(estimated explained variance) for the given model. Models with the addition of an extra covariate are given in columns to the right, with the rows signal feature combined into a model alongside either Alpha-theta, low beta or high beta power. For intra-nuclear analyses where there were two samples per subject the model involved a mixed design with random intercepts for each subject (GLMM). The P-value (P < 0.05; $\underline{\underline{\text{P < 0.01}}}$) for the significance of the predictor (row) in the presence of the covariate (column) is given alongside the approximate explained variance. Models in which both covariates are known to be significant independently but both lose significance when entered in a model are marked in red. In these cases, inference of predictor significance cannot be drawn due to collinearity between covariates. Results in bold indicate P-values that remain below the Bonferroni corrected decision level termed α^*^ (see section “Reporting of Statistics” in supplemental material). The number of tests considered is equal to total number conducted for each method [i.e., Power GLMM − 12 tests (1 f-test × 3 Bands × 2 Pairings + 6 multiple regressors models)].

Further analysis investigating the ON-OFF drug associated UPDRS difference demonstrated an effect in the low beta band [*r*_(9)_ = 0.827,ON-OFF *inter*] and this can be seen in the scatter plots in Figure 6E. However this effect was not found to be significant when entered into the GLM (*P* > 0.05, Ω^2^ = 0.390) suggesting that the within subject gradients are not consistent across the whole group.

### LFP/HPI cross correlation

In order to determine whether UPDRS correlations of power arose from increased movement artifact that may be associated with lower symptom scores we ran cross-correlations for alpha-theta band passed LFPs with that of the RMS fluctuation of the head movement (HPI) data as measured by the MEG scanner.

From the pooled cross correlation (Image 2 in Supplementary Material) it can be seen that the alpha-theta band envelope does not show significant correlation with the HPI RMS at any lags. This result does not provide evidence for a non-neuronal source of the measured activity in the alpha-theta band.

## Discussion

A table summarizing the results of the analyses presented is given in Table 8. Only results which survived correction for multiple comparisons are shown.

**Table 8:** **Summary of results from analysis with measures of synchronization in the STN**.

	**Alpha-theta**			**Low beta**			**High Beta**		
	**ON L-DOPA ANOVA**	**OFF UPDRS Correlation**	**ON-OFF UPDRS Correlation**	**ON L-DOPA ANOVA**	**OFF UPDRS Correlation**	**ON-OFF UPDRS Correlation**	**ON L-DOPA ANOVA**	**OFF UPDRS Correlation**	**ON-OFF UPDRS Correlation**
**POWER**									
Intra-nuclear		Negative		↓	Positive	Positive			
**COHERENCE**									
Intra-nuclear				↓	Positive				
Inter-nuclear									
**WPLI**									
Intra-nuclear					Positive				
Inter-nuclear									
**DFA-PS**									
Intra-nuclear									
Inter-nuclear					Positive	Positive			

Statistics are summarized for either the ON/OFF comparison of means with repeated measures ANOVA, OFF UPDRS correlation, or ON-OFF difference correlation for the three bands of interest. Only results with P-values that met the Bonferroni corrected decision level were included (see section “Reporting of Statistics” in supplemental material). Arrows for ON L-DOPA columns indicate the change of given measure from the ON to the OFF state. For OFF/ON-OFF UPDRS, Negative or Positive are displayed in order to show direction of correlation with the given measure.

### Limitations of the study

Due to need for invasive surgery it is not possible to conduct a similar experiment with neurologically healthy subjects. We do not know in healthy subjects what would be normal alpha-theta and beta power levels, coherence, WPLI or PS-DFA. Furthermore the signal characteristics themselves may be affected by surgery (stun effect), longevity and severity of the disease and chronic medication exposure. To control for this all measures have been performed within the same subject in different conditions-ON and OFF L-DOPA. Symptom severity is measured across subjects and the change in symptom severity with medication is measured within a subject initially and then correlated across subjects.

There is difficulty when comparing localization of recordings across patients. Despite confirmation of electrode targeting with post-operative imaging, variability of individual anatomy in the relatively small structure of the STN makes attributing anatomical location to functionality a challenge. As a consequence the attribution of neurophysiological phenomena to a particular neuroanatomical pathway or network should be viewed with caution.

As previously discussed, measurements of coherence are susceptible to zero-phase volume conduction effects. Whilst we can be confident that volume conduction is not a problem for inter-nuclear interaction, it is likely that volume effects are present within the region within and surrounding STN nuclei due to the close proximity of contacts on the electrode. Studies of volume conduction of LFPs demonstrate that the extent of the effect is complex and dependent on a number of factors such as source density, orientations, and the conducting media. This has led to a wide range of possible distances at which volume conduction may occur, ranging from 0.6 mm upwards to 5 mm (Kajikawa and Schroeder, 2011). The 2 mm contact separation distance for channels within the STN suggests that volume conduction is to be expected.

### Dopamine and spectral components of the STNr

#### STNr low beta power in parkinson's disease

We found that the amplitude of the power in low beta band is positively correlated with UPDRS clinical assessment of the severity of bradykinesia and rigidity symptoms (Figure 4B and Table 5). These findings suggest that the strength of 13–20 Hz oscillations within the STNr relate to severity of akinetic-rigid symptoms in PD.

We have reproduced the finding that beta power is reduced with treatment with L-DOPA (Priori et al., 2004; Weinberger et al., 2006; Hammond et al., 2007). The effect was found to occur robustly throughout the patient cohort and is evident in a large number of single channel spectra (for an example, see Figure 2C). There is also evidence for the subdivision of beta into high and low bands determined by their responsiveness to dopaminergic therapy such that low beta band is most affected by treatment with L-DOPA (see also Litvak et al., 2011). This effect is clear in the spectra shown in Figure 3A, in which two discrete peaks in beta range are seen - the low band is significantly modulated by dopamine, whilst high beta is not significantly changed.

We also note that changes in low beta power ON and OFF L-DOPA correlate with the scale of improvement of clinical symptoms with drug therapy. We show that stronger decreases in low beta power are associated with greater therapeutic benefit of L-DOPA. This effect is similar to that previously reported in Kühn et al. (2006) and strengthens the argument that reduction in beta power within STNr may be a good physiological target for treatments of PD.

#### STN alpha-theta power in parkinson's disease

In contrast to the findings for power in the low beta range, we report for the first time that the amplitude of alpha-theta power is negatively correlated with bradykinesia/rigidity symptoms. This suggests that increased alpha-theta power is associated with a less Parkinsonian motor state in untreated patients.

Alpha power in the STN was first reported by Priori et al. (2004) but these authors did not find it to be affected by L-DOPA. Later Alonso-Frech et al. (2006) reported alpha oscillations the power of which was raised in response to L-DOPA. Furthermore, they reported that the effect was disproportionately represented in patients exhibiting ON drug L-DOPA induced dyskinesias. In the present study we did not find a correlation between head movement and STNr alpha-theta activity. Thus we have no evidence for relation between alpha-theta activity and head movement during the STNr recording in the scanner.

The present findings are in agreement with Alonso-Frech et al. (2006) who found no connection between dyskinetic movements and STN alpha activity. This led the previous authors to conclude that alpha increase is not the result of involuntary movement *per se* but rather the physiological changes that allow dyskinesias to emerge. In future studies it may also be possible to study further the differential effects of alpha increase and beta decrease with improving Parkinsonian state through a more detailed analysis of the other symptoms of Parkinson's including gait impairment and postural control.

It has been reported that alpha activity correlates strongly with motor effort and to a lesser extent, reactivity in PD (Anzak et al., 2012). Alpha STN activity is also known to be coherent with a wider parietal-temporal network proposed to be tied to attentional functions (Litvak et al., 2011). Our findings here support the functional importance of alpha-theta band activity in PD.

### Sub-cortical connectivity within and across STNr

#### Intra-nuclear connectivity increases with severity of motor symptoms

Measurement of spectral coherence within STNr at rest has demonstrated that existence of a positive correlation between OFF state UPDRS and low beta intra-nuclear coherence. This result is maintained with WPLI analysis, thus supporting the hypothesis that measured connectivity is physiological and not the result of field spread. However, the positive relationship between increased UPDRS severity and increase functional connectivity measured with WPLI is statistically dependent on low beta power when regressing for UPDRS. This may be indicative of dispersion of extrinsically generated beta oscillations throughout locally coherent networks within the structures of the STN. This property has been theorized to allow the structure to form a global “switch” like response to cortical input (Gillies and Willshaw, 2004). It is possible that excess within-STNr coherence may subsequently result in a decreased sensitivity to cortical motor input as the structure is dominated by locally propagating beta oscillations. We found a correlation between increased UPDRS and strength of high beta coupling but again in a mixed regression model this effect was not independent of high beta power. Interestingly, previous studies looking at cortical-STN connectivity have suggested that activity in the high beta bands is more likely related to cortical communication (Oswal et al., 2013) although the role of this network in the pathophysiology of PD remains unclear.

#### Across hemisphere STN connectivity

Bilateral connectivity between STNrs is dominated by alpha-theta band coherence that is clearly visible as a peak in the coherence spectrum. This effect remains when correcting for volume conduction with the WPLI method although it is attenuated (Figure 5). This functional connectivity was not influenced by L-DOPA. Left-right alpha-theta band functional connectivity did not correlate with the severity of the motor off state.

In contrast to findings of de Solages et al. (2010) we did not find evidence of substantial left-right STN coherence in the beta range. Our results support those of Little et al. (2013) who found most left right STNr coherence in the alpha band. This finding of significant left-right alpha-theta band coherence complements the work of previous authors (de Solages et al., 2010; Hohlefeld et al., 2014; Kato et al., 2015) who found evidence for non-zero lag connectivity when using both standard coherence and the imaginary part of coherence in alpha-theta band. In contrast, we did not find evidence for increased bilateral alpha-theta or beta coherence in response to dopamine nor a relation to motor symptom severity. We suggest that a common drive to the left and right STNr may produce coupling although it is also possible that there is STNr to STNr connection. However, the actual anatomical connection between nuclei has yet to be elucidated (Little et al., 2013). Future work may be able to yield answers to these questions through the use of measures such as partial coherence to determine the existence of a common source, cortical or sub-cortical.

#### Zero lag connectivity

The reduction of the overall measure of signal correlation when using WPLI for both inter and intra-nuclear interactions when compared to standard coherence suggests that the contribution of zero-phase effects to the standard coherence is significant. This effect is interesting when considering inter-nuclear interactions as the relatively wide spatial separation of channels across a >26 mm wide commissure (Mavridis et al., 2013) indicates that the effects of volume conduction should be minimal. Zero phase lag interactions are entirely plausible biologically especially in the context of common synaptic input (Vicente et al., 2008; Gollo et al., 2014). It is suggested that there exist well-timed broadband cortico-subcortical projections that synchronize STN in both hemispheres via the hyper-direct pathway (Brunenberg et al., 2012). This view is supported by the finding of cortical leading coherence to the STN in the alpha band (Litvak et al., 2011). Our data lend support to the idea that there exists common input to both left and right STNr.

### Persistent temporal correlations are present in the phase coupled STN

Having identified the significant components of long-range synchrony via coherence and WPLI, as well as localized oscillations through analysis of the power spectrum, we next determined how the temporal structure of pairs of signals' phase interactions changed with the administration of dopamine and with the degree of patients' clinical motor symptoms. This is a novel approach to studying synchronization phenomena within the basal ganglia.

Our results demonstrate the existence of LRTCs in the phase dynamics of the coupled left and right STNr for alpha-theta and low beta bands such that non-trivial (>0.5) scaling exponents are measured and that the majority of fluctuation plots were deemed to be valid for power law scaling i.e. linear. We demonstrated that low beta band DFA-PS exponent magnitudes for inter-hemispheric pairs positively correlate with symptom severity in the Parkinsonian OFF state. Furthermore, low beta range DFA-PS exponents are predictors of UPDRS independently from power in the same band. The scaling statistics of phase dynamics detected by DFA-PS thus provide a novel way through which to characterize oscillations; one that is distinct from standard measures of power and coherence.

In the case of complete phase ordering there are no fluctuations of the derivative of phase difference and the corresponding DFA is uninterpretable. We measure this through accessing the linearity of the fluctuation plot. This method is outlined to in Section Assessing Fluctuation Plot Linearity. As the majority of exponents were validated with the described technique we can ascertain that there is a power-law (or at least long tailed) autocorrelation of the signal. This observation may suggest that there is a difference between PD ON and OFF states in the system's proximity to a critical bifurcation. Aburn et al. (2012) discuss the use of autocorrelation methodology to estimate a system's proximity to a super-critical Hopf bifurcation at the point limit cycle behavior emerges. At this point we speculate that measures of autocorrelation of phase difference (DFA-PS) will increase; in order to explore this further, modeling studies based on neural mass models such as that used by Aburn et al. (2012) are required to understand the interactions between phase and amplitude in the onset of synchrony.

The findings reported here suggest that the more severe the motor impairment of a patient, then the closer the bilateral subthalamic network is to the onset of synchronization (see (Botcharova et al., 2014) for model of synchronization onset). In the framework of the critical coupling hypothesis we would suggest that this implies an underlying shift of the STN network toward a supercritical regime from which pathological synchrony can more easily emerge. Such a regime would ultimately reduce the effective transfer entropy via phase (see (Barnett et al., 2013) for more details), reducing the encoding space available to the network by a recruitment of highly coherent yet informationally redundant neuronal units in the disease state (Hanslmayr et al., 2012). Previous use of DFA-PS in analyzing changes during movement at the level of the left and right motor cortices show that compared to the resting state, movement is associated with a decrease in exponent value (Botcharova et al., 2015). This would suggest that an increase in the degree of LRTCs present in the derivative of the phase difference between two signals is associated with an anti-kinetic state. This interpretation could apply to our results concerning interhemispheric STN connections.

It is interesting to note that whilst changes in linear metrics of bilateral STN functional connectivity (coherence and WPLI) did not correlate with symptom severity, the temporal ordering of phase as measured by DFA-PS did positively correlate. This suggests that the bilateral STN connection has greater susceptibility to entrainment by extrinsic drives such as that from the cortex or basal ganglia oscillators within the STN's local network. This may be indicative of a broader neuro-dynamic shift which increases the resonant properties of the basal ganglia to cortical inputs- a model which has recently been demonstrated to account for the generation of beta oscillations in PD (Pavlides et al., 2015).

We cannot completely exclude the possibility that the magnitude of beta oscillations may have affected the estimate of DFA-PS. We do however note the following: (i) DFA-PS exponents are independently correlated with the severity of bradykinetic symptoms in the OFF state, as estimated using the mixed regression models in Section Correlation of Connectivity Measures with OFF state Motor Symptom Severity and (ii) the effect of L-DOPA on alpha-theta and low beta power and DFA-PS is complex. L-DOPA administration tends to increase alpha-theta power and decrease low beta power; the corresponding changes in DFA-PS exponents in each frequency band do not mirror the power changes.

Our results demonstrate that in line with theoretical predictions: DFA-PS quantifies the propensity of the system to excessive synchronization which in turn relates to clinical impairment. However, in the specific case of pathological oscillations in PD, changes in power, particularly in the low beta band provide a clearer marker of these pathological processes over that of DFA-PS. Given that estimating power is more straightforward than that of DFA-PS exponent and can be done in real time, it does not seem to be the case that computing DFA-PS is a pragmatic way of inferring clinical state from STN LFP. However, when recordings from multiple parts of the cortico-basal ganglia circuit are available, DFA-PS might be useful for identifying those connections in the circuit that may modulate the system's susceptibility to pathological synchronization, especially its susceptibility toward synchronizing in response to external input. Performing this kind of analysis for cortico-subthalamic connections using MEG data collected from the patients included in the present paper will be the subject of future work and we will ask which changes in network connectivity can lead to pathological oscillations?

## Conclusions

Our study has added further detail to the description of oscillations and synchronous dynamics in the Parkinsonian brain. We report evidence for a contrasting effect of alpha-theta and low beta oscillation magnitude and their relation to L-DOPA treatment. Our findings strengthen the idea that beta oscillations act as an akinetic signal. Measures of linear association between signals (coherence and WPLI) emphasize the importance of beta oscillations in PD but in a GLMM they do not further explain Parkinsonian OFF clinical status. In contrast DFA-PSA exponent value adds to the explanation of the clinical status in Parkinsonian OFF. The scaling statistics of inter-nuclear interactions suggest that the network maybe closer to a transition to greater synchronization. These findings may help to explain why oscillations in the STN appear to be so important in determining patients' motor outcomes and they help us to understand further the mechanisms by which treatment such as L-DOPA and DBS achieve clinical improvement through modulation of neural synchrony.

## Author contributions

VL, AJ, and PB collected the data. TW analyzed the data. TF, PL, and LZ recruited patients for the study and collected clinical data. MB collected clinical data. VL, SF, and LB developed methods for data analysis. TW, SF, LB, and VL wrote the paper.

## Funding

TW receives funding from the Engineering and Physical Sciences Research Council (EPSRC grant ref: EP/M506448/1). The Wellcome Trust Centre for Neuroimaging is supported by core funding from the Wellcome Trust 091593/Z/10/Z. SF acknowledges funding support from the UCLH Biomedical Research Center.

### Conflict of interest statement

The authors declare that the research was conducted in the absence of any commercial or financial relationships that could be construed as a potential conflict of interest.
