# Supplementary material for: The Parkinsonian Subthalamic Network: Measures of Power, Linear, and Non-linear Synchronization and their Relationship to L-DOPA Treatment and OFF State Motor Severity
Source: Front Hum Neurosci. 2016 Oct 25;10:517. doi: 10.3389/fnhum.2016.00517 (PMC5078477; doi:10.3389/fnhum.2016.00517)
Supplement: Supplementary file 1 [file DataSheet1.docx]

# Appendix I

Model set used for PEB validation of linear fluctuation plots.

| *Linear* | $\boldsymbol{g}\left( \boldsymbol{x} \right)\boldsymbol{=ax+b}$ |
| --- | --- |
| *Quadratic* | $\boldsymbol{g}\left( \boldsymbol{x} \right)\boldsymbol{=a}\boldsymbol{x}^{\boldsymbol{2}}\boldsymbol{+bx+c}$ |
| *Cubic* | $\boldsymbol{g}\left( \boldsymbol{x} \right)\boldsymbol{=a}\boldsymbol{x}^{\boldsymbol{3}}\boldsymbol{+b}\boldsymbol{x}^{\boldsymbol{2}}\boldsymbol{+cx+d}$ |
| *Quartic* | $\boldsymbol{g}\left( \boldsymbol{x} \right)\boldsymbol{=a}\boldsymbol{x}^{\boldsymbol{4}}\boldsymbol{+b}\boldsymbol{x}^{\boldsymbol{3}}\boldsymbol{+c}\boldsymbol{x}^{\boldsymbol{2}}\boldsymbol{+dx+e}$ |
| *Quintic* | $\boldsymbol{g}\left( \boldsymbol{x} \right)\boldsymbol{=a}\boldsymbol{x}^{\boldsymbol{5}}\boldsymbol{+b}\boldsymbol{x}^{\boldsymbol{4}}\boldsymbol{+c}\boldsymbol{x}^{\boldsymbol{3}}\boldsymbol{+d}\boldsymbol{x}^{\boldsymbol{2}}\boldsymbol{+ex+f}$ |
| *Square-root* | $\boldsymbol{g}\left( \boldsymbol{x} \right)\boldsymbol{=}\boldsymbol{ax}^{\frac{\boldsymbol{1}}{\boldsymbol{2}}}\boldsymbol{+b}$ |
| *Cube-root* | $\boldsymbol{g}\left( \boldsymbol{x} \right)\boldsymbol{=}\boldsymbol{ax}^{\frac{\boldsymbol{1}}{\boldsymbol{3}}}\boldsymbol{+b}$ |
| *Logarithm* | $\boldsymbol{g}\left( \boldsymbol{x} \right)\boldsymbol{=a log(x)+b}$ |
| *Exponential* | $\boldsymbol{g}\left( \boldsymbol{x} \right)\boldsymbol{=a exp(x)+b}$ |

# Appendix II

### Removing Outliers

In order to correct for outliers in the signal features (power in band, coherence, WPLI and PS-DFA exponents) we opted to use a threshold set using the median absolute deviation (MAD) which is more robust to the influence of outliers than the more conventionally used standard deviation (Huber, 2004).

For a univariate series *x_1_,x_2_,..,x_i_ ,* the MAD is given by the median of the absolute residuals of the data from its median:

$$MAD=M\left( \left| x_{i}-M\left( x_{i} \right) \right| \right)$$

Where *x_i_* is the vector of data, and *M* is the median of the series. MAD is a consistent estimator of the standard deviation:

$$\hat{\sigma}=b\cdot MAD$$

Where b is a constant scale factor that is dependent upon the distribution and is set at *b* = 1.483 which assumes data is normally distributed. A decision criterion is then defined by setting a range from the median value using a set number of MADs:

$$M\left( x_{i} \right)-\theta\cdot b\cdot MAD<X_{i}<M\left( X_{i} \right)+\theta\cdot b\cdot MAD$$

Where the acceptance range is the median plus or minus a multiplier of the MAD (*θ)*. In this case *θ* was set to 2.5 which is set to give a conservative removal of outliers as recommended in Leys, Ley, Klein, Bernard, & Licata, (2013). When dealing with ON/OFF paired data outliers were removed independently for each set of features.

### Reporting of Statistics

Unless otherwise stated all tests of the data were computed with the outliers removed by the MAD procedure described in previous section and remaining degrees of freedom reported in the tables. When testing for significant differences between signal features in ON and OFF states, the samples were first tested for normality using a Shapiro-Wilk test. For the samples that were normally distributed a paired Student’s t-test was used, otherwise a non-parametric Kruskal-Wallis test was used to compare means. Tests were conducted only for pairs of data in which no outliers had been removed. Degrees of freedom for tests (denoted *d.f.*) are reported in brackets marking the test used as well as the 95% confidence interval for the difference in means. Correlations between features were computed using the non-parametric Spearman’s rank-order test and correlation *r* coefficients are reported along with their corresponding P-values. When correlations were deemed significant then linear least squares regressions were computed and their corresponding R^2^ reported.

All P-values were compared to a Bonferroni corrected set level *α^*^= α/K*, where *K* is the number of tests used for each method of analysis and *α* is the uncorrected decision point (*α*=.05). In the case of univariate measures (power, frequency) this was equal to 3 tests (3 bands) and for bivariate measures, 6 tests (3 bands x 2 pairings intra-/inter-) done for either ANOVA or Spearman correlations. GLMM tests were counted to include tests of alternative models with multiple covariates. Results which survived Bonferroni correction were marked in bold in the tables and discussion was limited to these results only.

### Confidence Intervals

Confidence intervals for power spectra and coherence spectra were computed using the analytic expressions given in (Halliday et al., 1995). Confidence limits for cross correlations are computed as in (Hanson & Yang, 2008) and are given by:

$$c_{1-\alpha}=\sqrt{2}\cdot Erfc^{-1}\left( \alpha\right)\frac{\sigma_{xy}}{\sqrt{n_{t}}}$$

where the pooled variance is:

$$\sigma_{xy}=\sqrt{\frac{N-1}{N^{2}}var\left\{ x \right\}var\{y\}}$$

where *N* is the number of samples in the signals of interest; *n_t_* is the number of lags included in the test; and *Erfc^-1‑^*is the inverse complementary error function for a given significance level *α*. All confidence intervals are given to level of *P* = .05.

### OFF Drug UPDRS Correlation with Signal Features

Clinical estimates of Parkinsonian bradykinesia and rigidity severity in the ON and OFF state were obtained by summing items 3.3 to 3.7 of the original UPDRS including assessment of arm and leg rigidity; finger tapping; hand and arm movements; and toe tapping (Fahn, Jenner, Marsden, & Teychenne, 1987). In the OFF state there was a large spread of UPDRS scores indicating differing clinical severity. These UPDRS scores along with the change in UPDRS score when going from OFF drug to ON drug were tested for correlation with the measures of neural dynamics. We performed pairwise correlation and regression analysis for each LFP measure (power, coherence WPLI and DFA-PS) with the clinical scores. In the case of bivariate measures, lateralized (intra-nuclear) pairings were correlated with contralateral hemi-body scores (scores from assessment of only one side of the body) and for inter-nuclear pairs we used the average score of both left and right assessments. We also correlated measures with clinical improvement as determined by correlating the ON-OFF difference in the clinical scores and the physiological measure of interest.

Results for power, coherence and WPLI when correlated with the clinical scores are shown in table 5 where the Spearman correlation coefficient is shown alongside the coefficient of determination (R^2^) for the corresponding linear regression. The P-value from the correlation is shown as well as the degrees of freedom.

### Mixed Modelling

In order to determine the predictive value of the metrics presented with respect to the UPDRS we employed a standard mixed-effects design for general linear modelling in SPSS which we will term GLMM. Using this we aimed to identify the unique contribution of a predictor (such as power, coherence or DFA-PS) to the explained variance of the response variable (UPDRS). As experimental design resulted in repeated measures within subjects (left and right samples we opted for a hierarchical model which incorporated group level *fixed effects* in which the effect is treated as a set quantity deviating around a mean; as well as *random effects* in which inter-subject variability is accounted for with a random intercept for each subject. This mixed modelling approach is adopted in order to control for dependencies of effects within and between subjects (Friston, Stephan, Lund, Morcom, & Kiebel, 2005).

In the report, we give P-values for significance tests of fixed effects (F-ratios) for covariates as well as the estimated explained variance for the complete model. The estimated explained variance, Ω^2^ is given by:

$$\Omega^{2}=1-\frac{var(r_{i}MIXED)}{var(r_{i}RANDOM)}$$

Where Ω^2^ is a value between 1 and 0 representing the fraction of explained variance; *var* indicates variance; *r­_I ­_*is the residual error for either the *MIXED* model with both random and fixed effects or the *RANDOM* model that has random effects only (Xu, 2003).

We aimed to access the relative predictive power of standard linear measures such as power and coherence in comparison to those potentially gained using DFA-PS. This was achieved by entering predictors into a multiple regression model and then testing for significance of their corresponding fixed effects when regressing for UPDRS. In the case where a predictor lost significance for its effect in the presence of another covariate we determined that the metric did not provide a significant increase in linear prediction of the UPDRS. For the inter-nuclear correlations (where the is only one sample per subject per condition) it was not possible to compute random effects and so a standard fixed effects GLM design was used.
