# Supplementary figures and images for: The Parkinsonian Subthalamic Network: Measures of Power, Linear, and Non-linear Synchronization and their Relationship to L-DOPA Treatment and OFF State Motor Severity
Source: Front Hum Neurosci. 2016 Oct 25;10:517. doi: 10.3389/fnhum.2016.00517 (PMC5078477; doi:10.3389/fnhum.2016.00517)

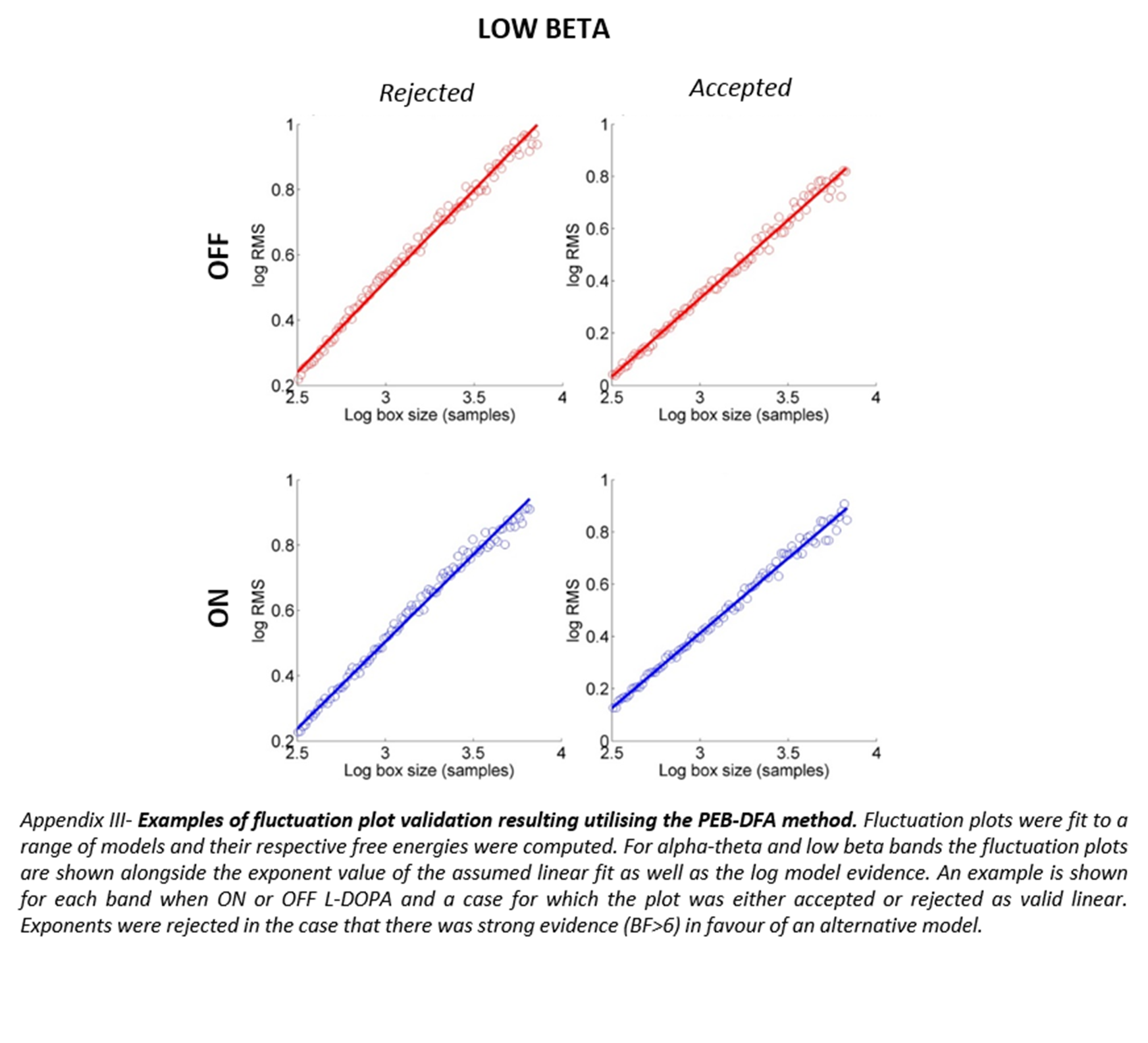

Supplement: Supplementary file 2 [file Image1.TIF]

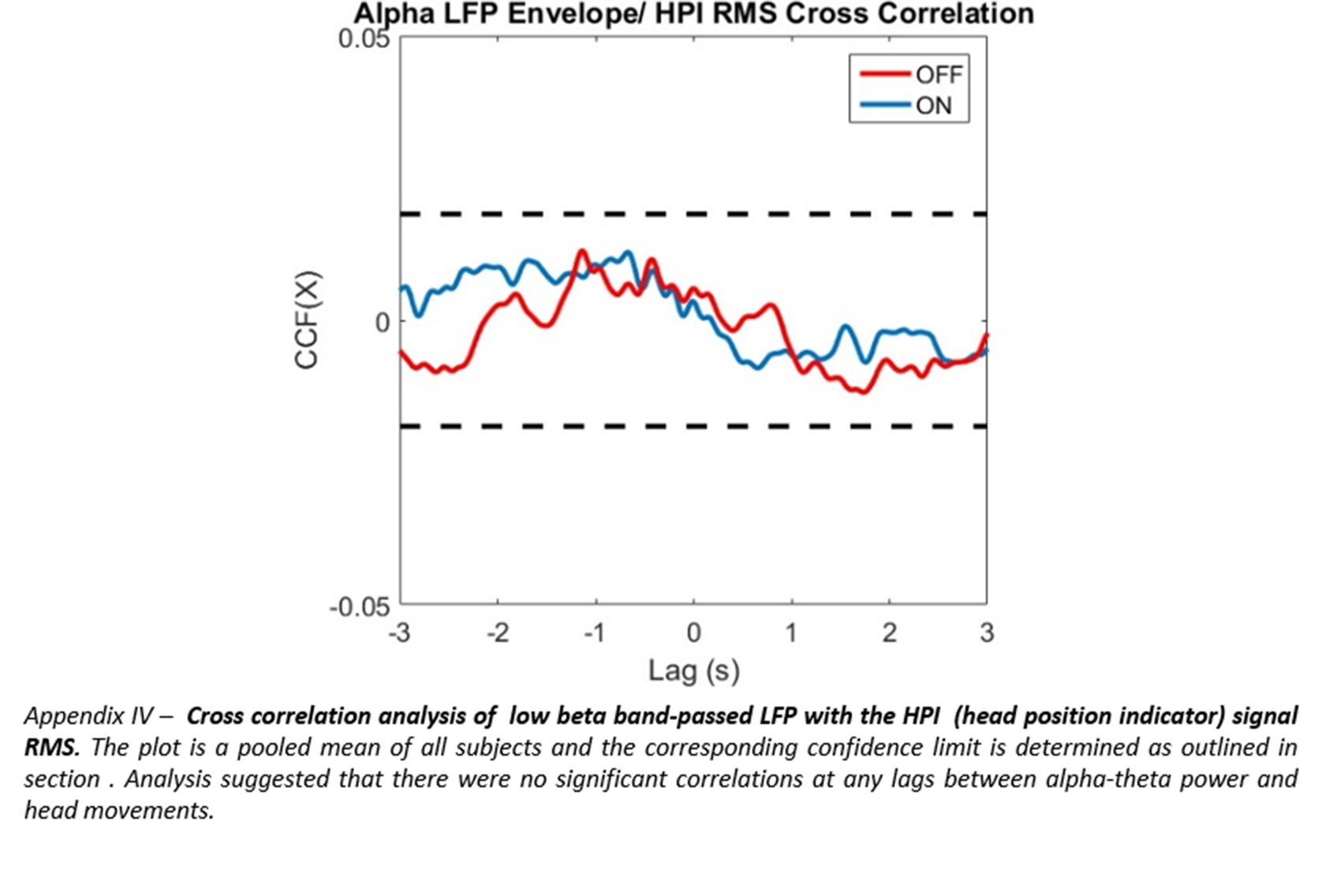

Supplement: Supplementary file 3 [file Image2.TIF]

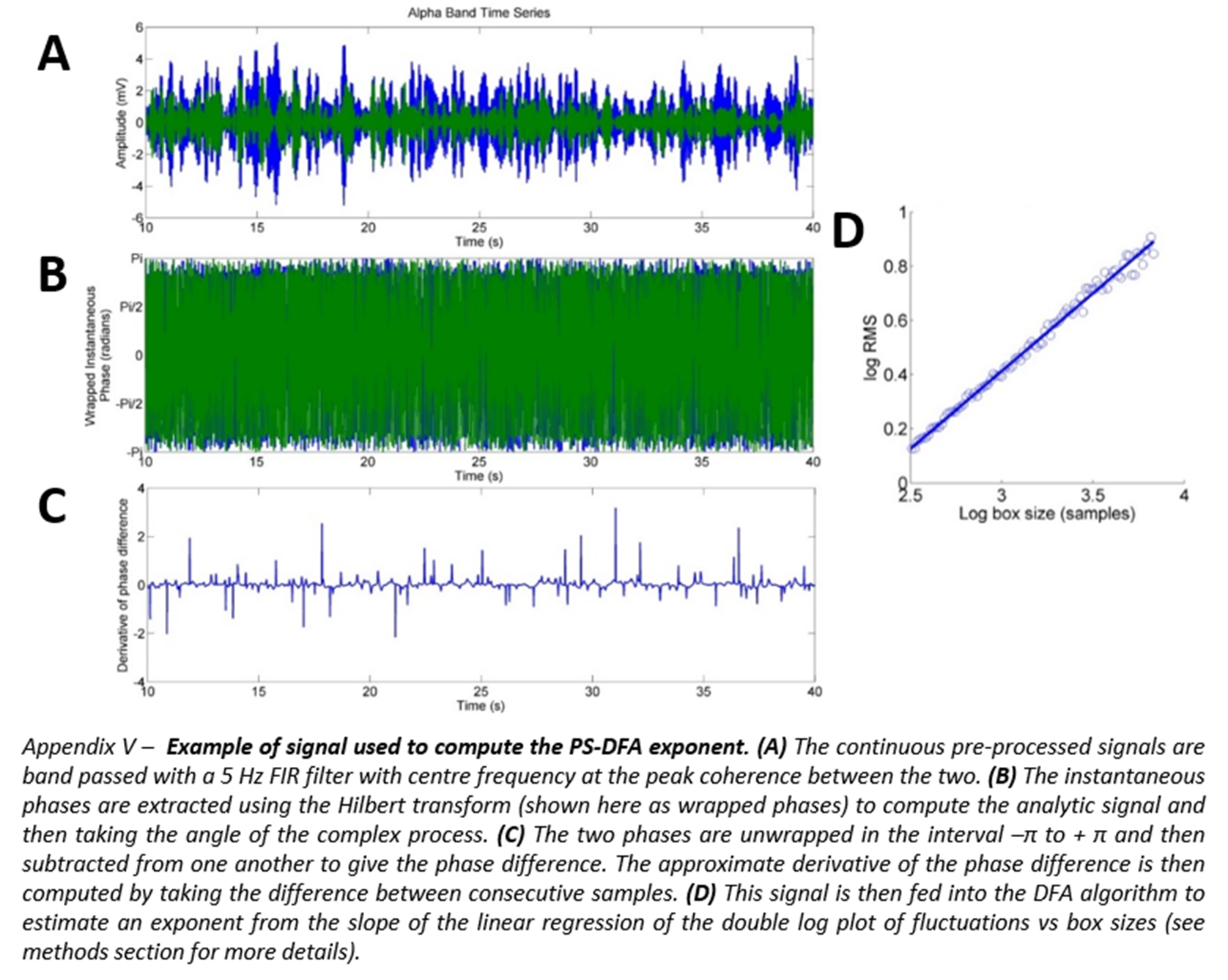

Supplement: Supplementary file 4 [file Image3.TIF]
